# Is a verification phase useful for confirming maximal oxygen uptake in apparently healthy adults? A systematic review and meta-analysis

**DOI:** 10.1371/journal.pone.0247057

**Published:** 2021-02-17

**Authors:** Victor A. B. Costa, Adrian W. Midgley, Sean Carroll, Todd A. Astorino, Tainah de Paula, Paulo Farinatti, Felipe A. Cunha

**Affiliations:** 1 Graduate Program in Exercise Science and Sports, University of Rio de Janeiro State, Rio de Janeiro, Brazil; 2 Laboratory of Physical Activity and Health Promotion, University of Rio de Janeiro State, Rio de Janeiro, Brazil; 3 Department of Sport and Physical Activity, Edge Hill University, Ormskirk, Lancashire, England; 4 Department of Sport, Health and Exercise Science, University of Hull, Hull, England; 5 Department of Kinesiology, California State University, San Marcos, California, United States of America; 6 Department of Clinical Medicine, Clinics of Hypertension and Associated Metabolic Diseases, University of Rio de Janeiro State, Rio de Janeiro, Brazil; University of Bourgogne France Comté, FRANCE

## Abstract

**Background:**

The ‘verification phase’ has emerged as a supplementary procedure to traditional maximal oxygen uptake (VO_2max_) criteria to confirm that the highest possible VO_2_ has been attained during a cardiopulmonary exercise test (CPET).

**Objective:**

To compare the highest VO_2_ responses observed in different verification phase procedures with their preceding CPET for confirmation that VO_2max_ was likely attained.

**Methods:**

MEDLINE (accessed through PubMed), Web of Science, SPORTDiscus, and Cochrane (accessed through Wiley) were searched for relevant studies that involved apparently healthy adults, VO_2max_ determination by indirect calorimetry, and a CPET on a cycle ergometer or treadmill that incorporated an appended verification phase. RevMan 5.3 software was used to analyze the pooled effect of the CPET and verification phase on the highest mean VO_2_. Meta-analysis effect size calculations incorporated random-effects assumptions due to the diversity of experimental protocols employed. I^2^ was calculated to determine the heterogeneity of VO_2_ responses, and a funnel plot was used to check the risk of bias, within the mean VO_2_ responses from the primary studies. Subgroup analyses were used to test the moderator effects of sex, cardiorespiratory fitness, exercise modality, CPET protocol, and verification phase protocol.

**Results:**

Eighty studies were included in the systematic review (total sample of 1,680 participants; 473 women; age 19–68 yr.; VO_2max_ 3.3 ± 1.4 L/min or 46.9 ± 12.1 mL·kg^-1^·min^-1^). The highest mean VO_2_ values attained in the CPET and verification phase were similar in the 54 studies that were meta-analyzed (mean difference = 0.03 [95% CI = -0.01 to 0.06] L/min, *P* = 0.15). Furthermore, the difference between the CPET and verification phase was not affected by any of the potential moderators such as verification phase intensity (*P* = 0.11), type of recovery utilized (*P* = 0.36), VO_2max_ verification criterion adoption (*P* = 0.29), same or alternate day verification procedure (*P* = 0.21), verification-phase duration (*P* = 0.35), or even according to sex, cardiorespiratory fitness level, exercise modality, and CPET protocol (*P* = 0.18 to *P* = 0.71). The funnel plot indicated that there was no significant publication bias.

**Conclusions:**

The verification phase seems a robust procedure to confirm that the highest possible VO_2_ has been attained during a ramp or continuous step-incremented CPET. However, given the high concordance between the highest mean VO_2_ achieved in the CPET and verification phase, findings from the current study would question its necessity in all testing circumstances.

**PROSPERO Registration ID:**

CRD42019123540.

## Introduction

Maximal oxygen uptake (VO_2max_) represents the upper physiological limit of the utilization of oxygen for producing energy during strenuous exercise performed until volitional exhaustion [1, 2]. The VO_2max_ is widely regarded as the gold standard measure of cardiorespiratory fitness and is typically determined using a cardiopulmonary exercise test (CPET) in clinical, applied physiology, and sport and exercise science settings [1, 3–6]. The VO_2max_ is often used to diagnose cardiovascular disease [7], predict all-cause mortality [8–10], develop exercise prescriptions [3, 11, 12], and evaluate the efficacy of exercise programmes [13–15]. Consequently, the validity of VO_2max_ values obtained during CPETs has widespread importance in clinical, sporting, and research-related contexts.

The use of indirect calorimetry for the determination of VO_2max_ during exercise testing to volitional exhaustion on a treadmill or cycle ergometer has become common during the past few decades [16–18]. This has largely been attributed to the development of fast-responding metabolic gas analyzers allowing the time-efficient acquisition of real-time, breath-by-breath, respiratory gas exchange and flow rate data during CPET [see 19 for a review]. These technological advances have contributed to a transition from the Douglas bag method and time-consuming discontinuous step-incremented protocols to more time-efficient continuous ramp or pseudo-ramp protocols for determining VO_2max_ [20–25]. Despite the considerable progress in the efficiency by which CPET can be conducted and evaluated, there is still much to be learned about the determination of VO_2max_ [2, 24–30]. One particularly problematic aspect has been the challenge in identifying a lack of VO_2max_ attainment due to inappropriate test protocols, premature fatigue, or poor participant motivation and lack of effort [31].

The concept of a VO_2max_ originated almost 100 years ago with the seminal works of Hill and colleagues [32, 33]. They proposed the existence of an individual upper limit or ‘ceiling’ of VO_2_ during maximal exercise, beyond which no further increase in VO_2_ occurs despite increasing work rate (WR) and higher metabolic demand. The primary criterion for confirming that a VO_2max_ has been elicited has historically been based on the occurrence of a VO_2_ plateau, commonly defined as a small or no increase in VO_2_ despite a continued increase in WR [34]. The landmark study of Taylor et al. [34] was the first to use a formal VO_2_ plateau criterion, which was defined as an increase in VO_2_ of less than 0.150 L/min (or ≤ 2.1 mL·kg^-1^·min^-1^, considering an average body mass of 72 kg from 115 male participants) in response to a specific discontinuous step-incremented protocol performed over 3–5 laboratory visits. Subsequent studies have often used the Taylor et al. [34] criterion or alternative thresholds to confirm the attainment of a VO_2_ plateau [see 29 for a review]. Since the widespread adoption of continuous short-duration and ramp-based CPET protocols, several studies have reported low incidences of the VO_2_ plateau [35–39]. The variability in VO_2_ plateau incidence has been attributed to differences in the criteria used for detecting the VO_2_ plateau [29, 40], VO_2_ sampling intervals [36, 41, 42], exercise modality [43], the warm-up prior to the CPET [44], type of CPET protocol [45–48], and various participant characteristics [49–51].

In the absence of a VO_2_ plateau, secondary VO_2max_ criteria based upon achievement of threshold values for the respiratory exchange ratio (RER), percentage of age-predicted maximal heart rate, post-exercise blood lactate concentration, and ratings of perceived exertion (RPE) have become commonly used to evaluate whether a true VO_2max_ has been attained [29, 40]. However, this approach has been widely criticized by numerous investigators due to the individual variability in maximal physiological responses for these variables and lack of specificity in identifying individuals who did not continue the CPET to their limit of exercise tolerance. Research has shown that some individuals can satisfy some of the secondary criteria thresholds long before the highest VO_2_ value observed in the CPET has been attained [2, 29, 37, 39]. The maximal RER criterion, for example, can be satisfied at VO_2_ values 27–39% lower than the highest VO_2_ value achieved in the CPET [37, 39]. Like the VO_2_ plateau, secondary VO_2max_ criteria are often dependent on exercise modality, test protocol, and participant characteristics [29].

A review by Midgley et al. [29] suggested a new set of standardized VO_2max_ criteria should be developed that are independent of exercise modality, test protocol, and participant characteristics, so they can be universally applied. In 2009, Midgley and Carroll [28] provided an early narrative review of an evolving test procedure that showed promise for developing more standardized VO_2max_ criteria, the so-called ‘verification phase’. The verification phase consists of an appended square wave bout of severe-intensity exercise (e.g. above critical power), or similar multistage exercise bout, performed until the limit of exercise tolerance [28]. It is commonly applied after a short recovery period from a CPET, however, longer recovery periods of up to 24–48 hours also have been used [52]. The verification phase is based on the premise that when the highest VO_2_ values in the CPET are consistent with the verification phase (typically within 2–3% in accordance with the test-retest reliability of VO_2max_), this provides substantial empirical support that the highest possible VO_2_ has been elicited. Poole and Jones [2] recently stated that to confirm the attainment of VO_2max_ a verification phase should be performed at a higher WR than the last load attained in the CPET (i.e. > WR_peak_) in all future studies. Conversely, Iannetta et al. [25] recommended WRs within the upper limit of the severe exercise intensity domain to allow the verification phase to be maintained long enough for VO_2max_ attainment. According to their recent findings, verification phases performed at 110% of the WR_peak_ attained during CPETs with increment rates of 25 and 30 W/min resulted in exercise durations that were too short to allow VO_2_ to reach the highest VO_2_ recorded at the end of the preceding ramp CPETs [25]. Along with exercise intensity and duration, it is also unclear whether other factors affect the utility of the verification phase such as exercise modality, differences in the type and duration of the recovery period between the verification phase and CPET, whether a verification criterion threshold is adopted, and participant characteristics such as sex and cardiorespiratory fitness levels.

Given the considerable uncertainty regarding the application of the verification phase, it is feasible to think that a systematic review and meta-analysis is needed to comprehensively summarize the evidence for improving our understanding of the strengths and weaknesses of the substantial number of different verification procedures that have been utilized and its impact on the attainment of VO_2max_. Thus, the aim of the present study was to systematically review and provide a meta-analysis on the application of the verification phase for confirming whether the highest possible VO_2_ has been attained during ramp or step-incremented CPETs in apparently healthy adults.

## Methods

### Protocol and registration

The systematic review was performed in accordance with the Preferred Reporting Items for Systematic Reviews and Meta-Analyses (PRISMA) guidelines. A completed PRISMA checklist is shown in S1 Checklist. The protocol for this study was recorded at http://www.crd.york.ac.uk/PROSPERO (CRD42019123540). The main questions addressed by the present study were: To what extent does the highest VO_2_ attained in the CPET differ from that attained in the verification phase? Secondly, are the highest VO_2_ values in the CPET and verification phase affected by the verification-phase characteristics (e.g. intensity, adoption of a criterion threshold, and aspects of the recovery period between the CPET and the verification phase), or even with respect to particular subgroups (e.g. sex, cardiorespiratory fitness levels, exercise test modality, and CPET protocol design) in apparently healthy adults?

### Search strategy

MEDLINE (accessed through PubMed), Web of Science, SPORTDiscus, and Cochrane (accessed through Wiley) were searched for peer-reviewed literature using a combination of medical subject heading (MeSH) descriptors, with a time frame that spanned the inception of each database until the search date (September 30^th^, 2020). The search strategy was developed based on the PICO method [i.e. *P*articipants: apparently healthy humans; *I*nterventions: any intervention involving exercise; *C*omparisons: incremental CPET and an appended square-wave or multistage verification phase; and *O*utcome: VO_2max_ confirmation]. The electronic search strategies for all databases are provided in S1 Text.

The terms were adapted for use with other bibliographic databases. Reference lists and citations of eligible articles were also hand searched for additional relevant studies. The search was performed in a standardized manner by two independent researchers (VABC and TP). Only English language studies were eligible for inclusion and only if they satisfied three *a priori* criteria: (1) involved apparently healthy participants who were ≥ 18 years of age; (2) determined VO_2max_ using expired gas analysis indirect calorimetry; and (3) the CPET was carried out using bipedal cycle ergometer or bipedal treadmill running or walking. Studies were excluded if they involved: (1) participants who had taken dietary supplements or drugs that could affect body mass, metabolic profile, or exercise performance; or (2) the use of non-maximal test protocols.

### Study selection

**Fig 1:**
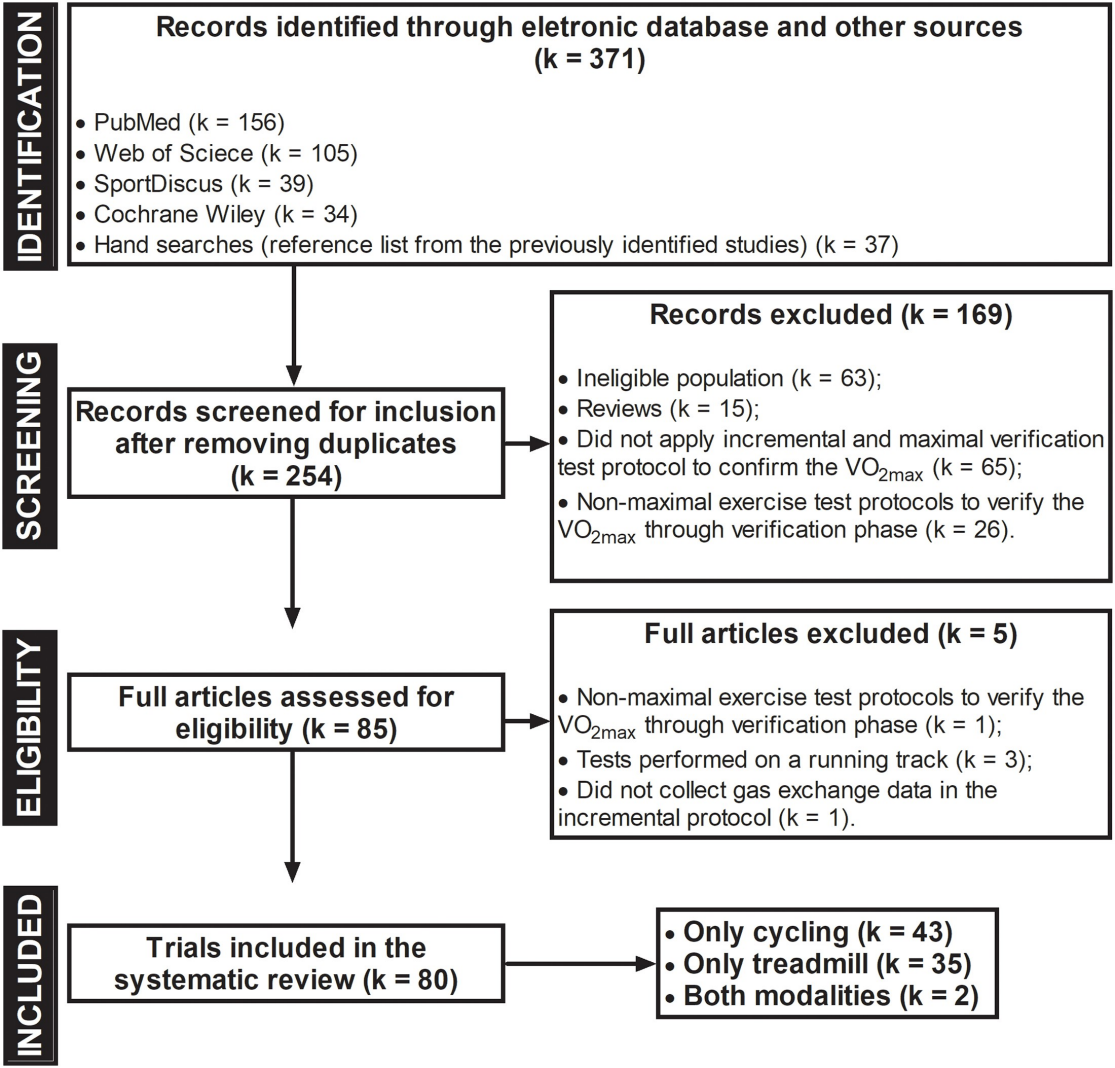
Flowchart of the systematic review and meta-analysis according to the PRISMA guidelines. VO_2max_: maximal oxygen uptake.

Potential studies were screened for inclusion using three methods: (1) title only; (2) title and abstract; and (3) full-text review. Two investigators independently searched and selected articles, and coauthors subsequently confirmed articles to be included in the analysis. Disagreements were resolved by consensus. Agreement between investigators with respect to inclusion and/or exclusion of potential trials was ratified in 252 randomly selected abstracts by means of Cohen’s kappa (κ = 0.811, *P* < 0.05). Fig 1 summarizes the screening and selection process.

### Data extraction and management

Two independent reviewers extracted data using a standardized form. The following data were summarized: (1) characteristics of study participants (total sample number, sex, age, body mass index [BMI], and cardiorespiratory fitness); (2) type of intervention (CPET and verification-phase duration, exercise modality, and exercise test protocol used); and (3) outcome measures (mean ± standard deviation [SD] for group VO_2max_ and protocol duration during the CPET and verification phase). Disagreements were resolved by consensus. When the relevant quantitative data were not reported, authors of the original studies were contacted to request the data.

### Quality assessment

The risk of bias for all eligible studies was not assessed because it does not apply to the characteristics of the present review. For example, randomization sequence generation and treatment allocation concealment were not applied, since there were no comparison groups and each individual acted as their own control. It is also noteworthy to mention the absence of blinding in both participants undergoing testing and evaluators who applied the CPET and verification phases, because procedurally all exercise protocols were performed in a fixed order (i.e. CPET followed by the verification phase). Given that VO_2max_ is the evaluation of an objective numerical variable, the blinding of the evaluator does not generate a different interpretation of the VO_2max_ values obtained in a CPET and verification phase. Finally, the assessment of incomplete outcome data (sample loss) and selective reporting of outcomes also does not apply, because it is a cross-sectional study with a single outcome of interest.

### Statistical analysis

All meta-analyses were performed using Review Manager (RevMan) software version 5.3 (Copenhagen, The Nordic Cochrane Centre, The Cochrane Collaboration, 2014). Data are presented as the mean ± SD unless otherwise stated. The outcome was the mean difference (95% confidence interval [CI]) between the CPET and verification phase for the highest absolute VO_2_ (L/min). Given that absolute VO_2_ are continuous data, the weighted mean difference (WMD) method was used for combining study effect size estimates. With the WMD method, the pooled effect estimate represents a weighted mean of all included study group comparisons. The weighting assigned to each individual study group (i.e. the comparison of the CPET and verification phase results) in the analysis is inversely proportional to the variance of the absolute VO_2_ (L/min). This method typically assigns more weight in the meta-analysis to studies with the highest precision (inverse variance) /larger sample sizes. The WMDs were calculated using random-effects models given the study group differences in CPET modalities and protocols, types of recovery, and verification phase protocols.

Heterogeneity of net study group changes in VO_2max_ (L/min) was examined using the Q statistic. Cochran’s Q statistic is computed by summing the squared deviations of each trial’s estimate from the overall meta-analytic estimate and weighting each trial’s contribution in the same manner as in the meta-analysis. *P*-values were obtained by comparing the statistic with a χ^2^ distribution with k-1 degrees of freedom (where k is the number of trials). A *P*-value of < 0.10 was adopted since the Q statistic tends to suffer from low differential power. The formal Q statistic was used in conjunction with the methods for assessing heterogeneity. The I^2^ statistic measures the extent of inconsistency among the results of the primary study groups, interpreted approximately as the proportion of total variation in point estimates that is due to heterogeneity rather than sampling error. Effect sizes with a corresponding I^2^ value of ≤ 50% were considered to have low heterogeneity. The publication bias of the articles was assessed using a funnel plot.

Subgroup analyses were defined *a priori* to investigate the magnitude of differences between CPETs and verification phases due to variations in sex, cardiorespiratory fitness level, exercise modality, CPET protocol design, or how the verification phase was performed. Forest plots were constructed to display values at the 95% confidence level. Effect sizes were calculated by subtracting the highest mean values for VO_2_ (L/min) observed in the CPET from the verification phase values, on the basis of grouping studies with selected verification-phase characteristics for intensity (i.e. sub *vs*. supra WR_peak_) and type of recovery between the CPET and verification phase (i.e. active *vs*. passive). The studies were also classified according to whether a criterion threshold for VO_2max_ was used for the verification phase (i.e. yes *vs*. no), whether the verification phase was performed in the same testing session as the CPET or on a different day, and the duration of the verification phase (i.e. ≤ 80 s, 81–120 s, and > 120 s). Stratified analyses were also conducted according to particular subgroups such as sex (i.e. male and female), cardiorespiratory fitness level using the cut-off points proposed by Astorino et al. [53] (i.e. low: < 40 mL·kg^-1^·min^-1^; moderate: 40–50 mL·kg^-1^·min^-1^; high: > 50 mL·kg^-1^·min^-1^), exercise test modality (i.e. cycling and running), and CPET protocol design (i.e. discontinuous step-incremented, continuous step-incremented, and ramp protocols).

## Results

The literature search identified 371 potential articles, with 334 obtained from electronic database searches and 37 from the wider inspection of reference lists and electronic citations of these articles. Eighty studies published between 1980 and 2020 met the eligibility criteria and were included in the systematic review (see Fig 1).

### Participants

The total number of participants recruited across all included studies was 1,680 (1,077 men, 473 women, and the sex of 130 participants was not specified). Included studies had a median (interquartile range [IQR]) sample size of 13 [10] participants. Participants were aged between 19 and 68 yr, all apparently healthy, and with a physical activity status ranging from sedentary to highly-trained endurance athletes. Thirty-six studies included only men, two included only women, 41 included both men and women, and one study did not specify the sex of the participants (see Table 1). On average, participants had a BMI within the normal range (mean ± SD [range]: 24.4 ± 2.5 [19.4–32.0] kg/m^2^) and a moderate level of cardiorespiratory fitness (VO_2max_ mean ± SD [range]: 46.9 ± 12.1 [23.9–68.6] mL·kg^-1^·min^-1^).

**Table 1:** Sample characteristics for studies that incorporated a cardiopulmonary exercise test (CPET) (k = 80).

Study				mean values		
	Population	Sex	N	Age	BMI	VO_2max_
		M/F		Years	kg/m^2^	mL·kg^-1^·min^-1^
Alexander and Mier [54]	Soccer players	M/F	5/6	21.3	22.7	57.7
Arad et al. [55]	Sedentary	M	19	33.4	25.8	30.0
		F	16	26.8	26.6	27.1
Astorino and DeRevere [56]	Recreationally trained	M/F	19/11	26	NS	47.2
		M/F	41/38	23.3	NS	40.5
Astorino and White [57]	Physically active	M	13	23.5	24.3	43.8
		F	17	22.9	22.0	40.7
Astorino et al. [53]	Low CRF	M/F	5/5	25.7	22.7	36.2
	Moderate CRF	M/F	5/5	26.3	24.1	46.4
	High CRF	M/F	9/1	26	23.7	57.9
Astorino et al. [58]	Active adults (HIIT-Baseline)	M/F	3/11	27	22	38.0
	Active adults (HIIT—Week 3)					40.4
	Active adults (Control—Baseline)	M/F	8/6	23	24	40.2
	Active adults (Control—Week 3)					40.5
Astorino et al. [59]	Active adults	M/F	14	27	22.5	38.0
Astorino et al. [60]	Sedentary	M/F	6/9	22.4	24.5	32.7
	Sedentary	M/F	1/8	21.8	22.9	42.1
Beltrami et al. [61]	Runners or cross-country skiers	M/F	23/3	29	23.5	61.3
Beltz et al. [62]	Recreationally trained	M	16	23.6	26.6	47.4
Bisi et al. [63]	Healthy adults	M	11	23.5	22.6	35.0
Chidnok et al. [64]	Active adults	M	7	20	24.8	57.7
Clark et al. [65]	Adults of various fitness levels	M/F	3/12	22	22.0	NS
Colakoglu et al. [66]	Athletes	M	9	24.2	23.0	59.7
Colakoglu et al. [67]	Well-trained athletes	M	9	23.6	23.1	60.2
Colakoglu et al. [68]	Athletes	M	9	23.6	23.1	60.2
Dalleck et al. [69]	Healthy adults	M/F	9/9	59.7	27.8	27.7
Day et al. [35]	Healthy adults	M	38	19–61	NS	NS
Del Giudice et al. [70]	Healthy adults	M	14	21.5	22.8	60.2
Dexheimer et al. [71]	Active adults	M	12	29	31.4	50.6
		F	5	25.6	24.4	43.7
Dicks et al. [72]	Firefighters	M	30	34.5	28.7	41.0
Dogra et al. [73]	Older adults (trained)	F	7	62.7	23.4	37.8
	Older adults (untrained)	F	10	68.8	26.1	24.1
Ducrocq et al. [74]	Recreationally trained	M/F	9/4	21.2	22.5	56.0
Elliott et al. [75]	Cyclists	M	8	40.5	25.2	53.7
Faulkner et al. [76]	Recreationally trained	M	13	25.5	24.5	63.9
Foster et al. [77]	Physically active non-athletes (cycling)	M	16	31.5	24.0	51.7
		F	4	28	21.6	
	Competitive runners (treadmill running)	M	12	21.6	22.9	56.3
		F	8	21	20.5	
Freeberg et al. [78]	Healthy adults	M/F	17/13	21.7	23.7	49.9
Goodall et al. [79]	Cyclists	M	9	28.1	23.1	61.1
Hanson et al. [80]	Recreationally trained	M/F	8/5	24	24.7	56.2
Hawkins et al. [81]	Distance runners	M/F	36/16	NS	NS	63.3
Hogg et al. [82]	Highly trained	M	14	28	23.2	68.6
Iannetta et al. [25]	Recreationally trained	M/F	6/5	28	21.8	52.6
James et al. [83]	Squash players	M/F	6/2	20.3	22.1	48.8
Jamnick et al. [84]	Trained cyclists	M	17	36.2	24.1	62.1
Jamnick et al. [85]	Active adults	M	31	29	25.2	48.6
		F	26	27	23.4	39.8
Johnson et al. [86]	Recreationally trained runners and cyclists	M/F	6/5	22	24.1	46.9
Keiller and Gordon [87]	Recreationally trained	M/F	9/2	22.4	24.4	51.6
Kirkeberg et al. [88]	Recreational-trained men	M	12	29	27.5	49.2
Knaier et al. [89]	Athletes	M	10	27.5	23.1	61.1
		F	7	28.4	22.5	54.3
Knaier et al. [90]	High cardiorespiratory fitness	M	8	27.4	22.8	62.8
		F	5	27.6	22.7	55.2
Kramer et al. [91]	Soccer players	M	15	23.1	23.0	50.5
Mann et al. [92]	Runners	M	20	30	24.2	60.2
		F	12	28	21.7	51.9
Mann et al. [93]	Runners	M	8	36	24.1	57.9
		F	2	32	24.9	49.9
Mauger et al. [94]	Well-trained runners	M	14	22.7	23.4	64.4
McGawley [95]	Recreational runners	M/F	5/5	32	NS	59.8
McKay et al. [96]	Healthy adults	M	12	25	NS	44.5
Midgley et al. [39]	Runners	M	10	39.3	23.6	53.6
	Cyclists	M	10	36.0	23.2	57.7
Midgley et al. [97]	Middle- and long-distance runners	M	16	38.7	23.0	57.1
Midgley et al. [98]	Distance runners	M	9	38.2	24.6	55.0
Mier et al. [99]	College athletes	M/F	8/27	20	23.5	55.5
Murias et al. [100]	Younger adults	M	30	25	24.9	49.4
	Older adults	M	31	68	25.8	33.0
Murias et al. [101]	Older adults	F	6	69	27.0	23.9
	Younger adults	F	8	25	23.8	41.2
Murias et al. [102]	Older adults	M	8	68	26.0	28.3
	Younger adults	M	8	23	25.2	48
Nalcakan [103]	Healthy adults	M	15	21.7	25.0	40.3
Niemela et al. [104]	Healthy adults	M	16	25–35	23.3	42.5
Niemeyer et al. [105]	Physically active	M	24	26.2	24.2	49.8
Niemeyer et al. [106]	Recreationally trained	M	46	25.6	24.0	50.8
Nolan et al. [107]	Active adults	M/F	6/6	23	22.7	57.5
Poole et al. [37]	Healthy adults	M	8	27	NS	50.8
Possamai et al. [108]	Recreationally trained cyclists	M	19	23	25.3	48.0
Riboli et al. [109]	Soccer players	M	16	22.5	22.4	59.2
Rossiter et al. [38]	Healthy adults	M	7	26	25.1	51.5
Sabino-Carvalho et al. [110]	Runners	M	14	22.3	21.2	67.0
		F	4	24	20.4	60.1
Scharhag-Rosenberger et al. [111]	Healthy adults	M/F	20/20	24	23.0	50.0
Scheadler and Devor [112]	Experienced runners	NS	13	25	22.5	64.9
Sedgeman et al. [113]	Recreationally trained	M/F	6/7	29	23.9	50.1
Stachenfeld et al. [114]	Healthy adults	M/F	33/18	30.6	NS	49.2
Straub et al. [115]	Trained cyclists	M	12	33	24.8	56.5
		F	4	38	22.1	
Strom et al. [116]	Healthy adults	M/F	21/29	30.3	24.0	47.3
Taylor et al. [117]	Runners and triathlon athletes	M	11	28.5	22.6	63.7
		F	8	26.3	21.8	52.3
Tucker et al. [118]	Nonexercise-trained youth	M	17	27	25.6	41.6
Vogiatzis et al. [119]	Cyclists	M	11	38	22.1	62.0
Weatherwax et al. [120]	Sedentary adults	M	5	53.6	32.0	32.3
		F	11	52.2	29.4	24.8
Weatherwax et al. [15]	Sedentary adults (standardized—baseline)	M/F	4/16	51.2	29.6	24.3
	Sedentary adults (standardized—week 4)	M/F	4/16	51.2	29.7	25.0
	Sedentary adults (standardized—week 8)	M/F	4/16	51.2	29.6	26.3
	Sedentary adults (standardized—week 12)	M/F	4/16	51.2	29.6	26.3
	Sedentary adults (individualized—baseline)	M/F	5/14	44.9	27.2	29.5
	Sedentary adults (individualized—week 4)	M/F	5/14	44.9	27.2	31.1
	Sedentary adults (individualized—week 8)	M/F	5/14	44.9	27.1	31.3
	Sedentary adults (individualized—week 12)	M/F	5/14	44.9	27.0	32.8
Weatherwax et al. [121]	Sedentary adults (control—baseline)	M/F	2/6	45.6	25.5	28.4
	Sedentary adults (control—week 12)				25.5	27.7
	Sedentary adults (standardized—baseline)	M/F	4/16	51.2	29.6	24.3
	Sedentary adults (standardized—week 12)				29.6	26.0
	Sedentary adults (individualized—baseline)	M/F	5/14	44.9	27.1	29.5
	Sedentary adults (individualized—week 12)				26.8	32.8
Weatherwax et al. [122]	Elite endurance-trained	M	18	21.9	19.8	62.8
		F	6	20.2	19.4	51.7
Wilhelm et al. [123]	Healthy adults	M	9	25	25.1	41.0
Williams et al. [124]	Healthy adults	M	8	27	NS	43.0
		M	5	23	NS	48.0
Wingo et al. [125]	Healthy adults	M	9	25	22.4	61.2
Yeh et al. [126]	Healthy adults	M/F	14/1	23.3	21.9	48.9

**Abbreviations:** *BMI* = body mass index; *CRF* = cardiorespiratory fitness level; *F* = female; *HIIT* = high-intensity interval training; *M* = male; *NS* = not stated; *VO*_*2max*_ = maximal oxygen uptake. *Note*: Whenever possible, authors were contacted to provide unpublished data.

### Characteristics of studies regarding the CPET and verification phase protocols to evaluate VO_2max_

Table 2 summarizes the characteristics of the CPET and verification phase protocols of the 80 studies included in this systematic review. Forty-three studies (54%) performed the CPET on a cycle ergometer, 35 (44%) on a treadmill, and two studies (3%) used both modalities. Seventy-three studies (91%) used continuous step-incremented or ramp/pseudo-ramp CPET protocols. Three (4%) used only discontinuous step-incremented protocols. Two studies (3%) used both discontinuous and continuous step-incremented protocols and another two studies (3%) applied self-paced protocols. Thirty-three (41%) of the 80 studies included in the review used one or more VO_2_ plateau or secondary VO_2max_ criteria to confirm the attainment of VO_2max_. Thirty studies used the VO_2_ plateau, 21 used the heart rate plateau or a criterion based on age-predicted maximal heart rate, 18 used the maximal RER attained in the CPET (RER_max_), and 8 used the post-CPET blood lactate concentration.

**Table 2:** Characteristics of the cardiopulmonary exercise test (CPET) and verification phase protocols used in the reviewed studies (k = 80).

Study	VO_2_ data sampling method	Traditional VO_2max_ criteria adopted	Exercise Modality	CPET Protocol	Recovery Phase Protocol	Verification Phase (VP) Protocol	Verification Criteria Threshold
Alexander and Mier [54]	30-s time average	VO_2_ plateau of 2.1 mL·kg^-1^·min^-1^	TR	CSI	10-min walking	1^st^ min: ↑ WR until matching the final stage of CPET; then ↑ slope to 2.5% and encouraged to running for 2-min	NS
				DisCSI			
Arad et al. [55]	20-s rolling mean	VO_2_ plateau (linear portion of the VO_2_-WR relationship); RER_max_ ≥ 1.10; ≥ 95% APMHR	CYC	Ramp	10-min active and 2–3 min passive	100% WR_peak_	NS
Astorino and DeRevere [56]	2×15-s	NS	CYC	Ramp	8-min active	105% WR_peak_	CPET *vs*. VP: VO_2max_ difference ≤ 3.0% and 3.3% and HR_max_ ≤ 4 bpm
					10-min active	110% WR_peak_	
Astorino and White [57]	15-s time average	NS	CYC	CSI	10-min active	one stage > CPET-Stage_final_	CPET *vs*. VP: VO_2max_ difference ≤ 3% and HR_max_ ≤ 4 bpm
Astorino et al. [53]	2×15-s	NS	CYC	Ramp	8-min active	2-min at 40–45% WR_peak_ and then 105% WR_peak_	CPET *vs*. VP: VO_2max_ difference < 2 mL·kg^-1^·min^-1^
Astorino et al. [58]	30-s time average	NS	CYC	Ramp	10-min active	105% WR_peak_	NS
Astorino et al. [59]	30-s time average	NS	CYC	CSI	10-min active	105% WR_peak_	VO_2max_ identified as the average of CPET and VP values
Astorino et al. [60]	2×15-s	NS	CYC	Ramp	≥ 24h	105%WR_peak_ reached in the CPET	NS
	30-s time average				1–1.5h	115%WR_peak_ reached in the CPET	
Beltrami et al. [61]	30-s intervals	VO_2_ plateau (difference between modelled and actual value >50% of the regression slope for the linear portion of the VO_2_-WR relationship—an average of 1.7 mL·kg^-1^·min^-1^)	TR	CSI (control)	15-min active or passive (self-choose: walk, jog or rest)	1^st^ min at 10 km/h (5% slope) and then ↑ 1 km/h > CPET-Speed_peak_	CPET *vs*. VP: VO_2max_ difference ≤ 123 ± 18 mL/min (or 1.7 mL·kg^-1^·min^-1^)
				CSI (reverse)			
Beltz et al. [62]	2×15-s	NS	TR	SPV	20-min passive	2-min at 30% CPET-WR_peak_, 1-min at 40–45% CPET-WR_peak_ and then until exhaustion at 105% CPET-WR_peak_	CPET *vs*. VP: VO_2max_ difference ≤ 3%
				Ramp			
Bisi et al. [63]	25-s moving-average	VO_2_ plateau (increase < than 3% or 2.1 mL·kg^-1^·min^-1^ between 2 steps of increment); RER_max_ ≥ 1.08 or 1.15; HR_max_ within 10 bpm of APMHR	CYC	CSI	6-min active	at least 3 min of cycling at 105% of the WR_peak_	CPET *vs*. VP: VO_2max_ difference ≤ 3%
Chidnok et al. [64]	30-s rolling-mean	NS	CYC	Ramp	Different day	See the formula for a proper reporting 3-min of ’all-out’ cycling	NS
Clark et al. [65]	15-s time average	NS	CYC	CSI	3-min active	WR_peak_ minus 2 stages	NS
Colakoglu et al. [66]	30-s average	VO_2_ plateau of 150 mL/min; RER_max_ ≥ 1.10; ≥ 90% APMHR;	CYC	Ramp	Different day	100% WR_peak_	NS
Colakoglu et al. [67]	30-s average	VO_2_ plateau of 150 mL/min; RER_max_ ≥ 1.10; HR_max_ within 10 bpm of APMHR; RPE ≥?	CYC	CSI	Different day	100% WR_peak_	VO_2_ plateau of 150 mL/min; RER_max_ ≥ 1.10; HR_max_ within 10 bpm of APMHR; RPE ≥?
Colakoglu et al. [68]	30-s average	VO_2_ plateau of 150 mL/min; RER_max_ ≥ 1.10; ≥ 90% APMHR; RPE ≥ 19–20	CYC	CSI	Different day	100%, 105%, and 110% WR_peak_ to attain the highest VO_2peak_ value	VO_2_ plateau of 150 mL/min; RER_max_ ≥ 1.10; ≥ 90% APMHR; RPE ≥ 19–20
Dalleck et al. [69]	2×15-s	NS	CYC	CSI	60-min passive	2-min at 50 Watts; then increased 105% WR_peak_	CPET *vs*. VP: VO_2max_ difference ≤ 3% and HR_max_ ≤ 4 bpm
Day et al. [35]	30-s time average	NS	CYC	CSI	Different day	90% WR_peak_ reached in the CPET	NS
Del Giudice et al. [70]	30-s time average	NS	TR	CSI	10-min passive	0.8 km/h > CPET-Speed_peak_	NS
Dexheimer et al. [71]	2×15-s	NS	TR	Pseudo-ramp protocol	5-10-min active	105% WR_peak_	CPET *vs*. VP: VO_2max_ difference ≤ 3%
Dicks et al. [72]	15-s time average	NS	TR	Pseudo-ramp protocol	3-min active	WR_peak_ minus 2 stages	NS
Dogra et al. [73]	every 20 ms	NS	CYC	Ramp	Different day	85%WR_peak_ reached in the CPET	NS
Ducrocq et al. [74]	breath-by-breath	NS	TR	CSI	5-min passive	105% WR_peak_	NS
Elliott et al. [75]	10-s epochs	NS	CYC	CSI	60-min	110%WR_peak_ reached in the CPET	NS
Faulkner et al. [76]	20-s time average	VO_2_ plateau of 2 mL·kg^-1^·min^-1^; RER_max_ ≥ 1.10; RPE ≥ 17; HR_max_ within 10 bpm of APMHR; La_max_ ≥ 8 mmol	TR	CSI	15-min passive	↑ speed over a 30-second period up to a 1 km/h > CPET-Speed_peak_	NS
Foster et al. [77]	30-s time average	rate of increase in VO_2_ during the last min < 50% when compared to the mid portion of the test	CYC	CSI	1-min active	25 Watts > CPET-WR_peak_	NS
			TR	CSI	3-min active	1.6 km/h > CPET-Speed_peak_ or 0.8 km/h if in the non-athlete group	
Freeberg et al. [78]	2×15-s	NS	TR	Incline-based protocol	10-min active	110% WR_peak_	CPET *vs*. VP: VO_2max_ difference ≤ 3%
Goodall et al. [79]	30-s mean	NS	CYC	CSI	5-min passive	as described by [38]; however, the intensity was not stated (i.e. 95 or 105%WR_peak_ reached in the CPET)	NS
Hanson et al. [80]	15-breath moving average	VO_2_ plateau of 2 mL·kg^-1^·min^-1^; RER_max_ ≥ 1.10	TR	CSI	10-min active	one stage > CPET-WR_peak_	CPET *vs*. VP: VO_2max_ difference ≤ 50 mL/min
Hawkins et al. [81]	40-s Douglas bag collection	NS	TR	CSI	Different day	130% WR_peak_	NS
Hogg et al. [82]	30-s time average	VO_2_ plateau (difference between modelled and actual value > 50% of the regression slope for the linear portion of the VO_2_-WR relationship); RER_max_ ≥ 1.10; RPE ≥ 17; HR_max_ within 10 bpm of APMHR	TR	CSI	10-min active (walking around the laboratory and stretching)	↑ speed over a 30-second period up to a speed stage > CPET-Stage_final_	CPET *vs*. VP: VO_2max_ difference ≤ 3%
				Incline-based SPV		speed halfway between speed_peak_ from the SPV_incline_ *vs*. predicted verification-stage speed of the CSI protocol	
				Speed-based SPV		speed halfway between speed_peak_ from the SPV_speed_ *vs*. predicted stage speed of the CSI protocol	
Ianetta et al. [25]	20-s rolling mean	VO_2_ plateau (linear portion of the VO_2_-WR relationship)	CYC	Ramp	10-min	110% WR_peak_	CPET *vs*. VP: VO_2max_ difference ≤ 0.1 L/min
James et al. [83]	10-s average	VO_2_ plateau of 2 mL·kg^-1^·min^-1^	TR	CSI	5-min active	↑ 1% > CPET-Slope	VO_2_ plateau of 2 mL·kg^-1^·min^-1^
Jamnick et al. [84]	20-s average	NS	CYC	CSI_1_ (1-min stage length)	5-min passive	90% WR_peak_—CSI_1_	NS
				CSI_3_ (3-min stage length)			
				CSI_5_ (5-min stage length)			
				CSI_7_ (7-min stage length)			
				CSI_10_ (10-min stage length)			
Jamnick et al. [85]	15-s time average	NS	CYC	CSI	3-min active	mean WR_peak_ minus 2 stages	CPET *vs*. VP: VO_2max_ difference ≤ 1.5 mL·kg^-1^·min^-1^ (or 3% CV)
Johnson et al. [86]	15-s intervals	NS	CYC	CSI	3-min active (50%WR_peak_)	WR_peak_ minus 2 stages	CPET *vs*. VP: VO_2max_ difference ≤ 3%
Keiller and Gordon [87]	30-s intervals	VO_2_ plateau (increase < than 50 or 100 mL/min) and HR plateau (increase < than 2 or 4 bpm) over the final two consecutive 30 s sampling periods	TR	CSI (Trials 1 and 2)	6-min passive	10 (female) and 9 (male) km/h and the ↑ 1% > CPET-Slope	CPET *vs*. VP: HR_max_ difference ≤ 2 or ≤ 4 bpm
Kirkeberg et al. [88]	30-s time average	NS	TR	CSI (short-term)	3-min active	CPET-Speed_end_ minus 2 stages, where stages were derived using specific equation	NS
				CSI (middle-term)			
				CSI (large-term)			
Knaier et al. [89]	30-s time average	RER_max_ ≥ 1.10; ≥ 95% APMHR; RPE ≥ 19; La_max_ ≥ 8 mmol	CYC	CSI	10-min active	2 min at 50% WR_peak_, 1 min at 70% WR_peak_, and then 1 stage > CPET-WR_peak_	CPET *vs*. VP: VO_2max_ difference ≤ 3%
Knaier et al. [90]	30-s time average	RER_max_ ≥1.05, 1.10 and 1.15; 90, 95 and 100% APMHR; RPE ≥ 19 and = 20; La_max_ ≥ 8 and 10 mmol	CYC	CSI	10-min active	2 min at 50% WR_peak_, 1 min at 70% WR_peak_, and then 1 stage > CPET-WR_peak_	CPET *vs*. VP: VO_2max_ difference ≤ 3%
Kramer et al. [91]	30-s intervals	NS	TR	CSI	3-min active	2 stages < CPET-WR_peak_	CPET *vs*. VP: VO_2max_ difference ≤ 3%
Mann et al. [92]	15-s	NS	TR	CSI	8-10-min	0.5 km/h > CPET-Speed_peak_	NS
Mann et al. [93]	15-s	NS	TR	CSI	8-10-min	0.5 km/h > CPET-Speed_peak_	NS
Mauger et al. [94]	5-s time average	VO_2_ plateau (increase < than 1.8 mL·kg^-1^·min^-1^ between 2 steps of increment); RER_max_ ≥ 1.10; HR_max_ within 10 bpm of APMHR; RPE ≥ 17; La_max_ ≥ 8 mmol	TR	CSI	10-min active	one stage > the last completed stage of the CPET	CPET *vs*. VP: VO_2max_ difference ≤ 1.8 mL·kg^-1^·min^-1^
McGawley [95]	30-s time average	VO_2_ plateau (increase < than 3% or 2 mL·kg^-1^·min^-1^ between 2 steps of increment); RER_max_ ≥ 1.15; HR_max_ within 10 bpm of APMHR; La_max_ ≥ 8 mmol	TR	CSI	9-min passive	105% at CPET-WR_peak_ (Trials 1 to 5)	CPET *vs*. VP: VO_2max_ difference ≤ 3%
McKay et al. [96]	15-s time average	NS	CYC	Ramp	5-min active	105%WR_peak_ reached in the CPET	NS
Midgley et al. [39]	30-s time average	VO_2_ plateau (difference between modelled and actual value > 50% of the regression slope for the linear portion of the VO_2_-WR relationship)	CYC	CSI	10-min passive	2 min at 50% WR_peak_, 1 min at 70% WR_peak_, and then 1 stage > CPET-WR_peak_, 2 min at 50% WR_peak_, 1 min at 70% WR_peak_, and then 1 stage > CPET-WR_peak_	CPET *vs*. VP: modelled and verification VO_2_ difference > 50% of the regression slope of the individual VO_2_-WR relationship; HR_max_ ≤ 4 bpm
			TR				
Midgley et al. [97]	30-s time average	absolute plateau in VO_2_; RER_max_ ≥ 1.10; HR_max_ within 10 bpm of APMHR	TR	CSI	10-min active	0.5 km/h > CPET-Speed_peak_	CPET *vs*. VP: VO_2max_ difference ≤ 2% and HR_max_ ≤ 2 bpm
Midgley et al. [98]	15 and 30-s time average	NS	TR	CSI 1-min stages	5-min passive	one stage > CPET	NS
				DisCSI 2-min stages			
				DisCSI 3-min stages			
Mier et al. [99]	30-s	VO_2_ plateau (2 mL·kg^-1^·min^-1^ and ≤ SD of the expected increase); RER_max_ ≥ 1.05, 1.10 and 1.15; ≥ 85% APMHR and HR_max_ within 10 bpm of APMHR	TR	CSI	10-min active (walking at slow pace)	intensity gradually increased over 2-min until match CPET-WR_peak_; after 1 min, the slope was increased 2.5% to running for 2-min	CPET *vs*. VP: VO_2max_ difference ≤ 2.2 mL·kg^-1^·min^-1^
Murias et al. [100]	20-s average time	NS	CYC	Ramp	5-min active	85% WR_peak_	CPET *vs*. VP: VO_2max_ difference ≤ 2.0 mL·kg^-1^·min^-1^
						105% WR_peak_	
Murias et al. [101]	20-s	NS	CYC	Ramp	5-min active	85%WR_peak_ reached in the CPET	NS
Murias et al. [102]	20-s	NS	CYC	Ramp	5-min active	85%WR_peak_ reached in the CPET	NS
Nalcakan [103]	30-s	VO_2_ plateau; RER_max_ ≥ 1.20; ≥ 90% APMHR	CYC	CSI	Different day	100% WR_peak_	NS
Niemela et al. [104]	every min	VO_2_ plateau (≤60 mL/min for men and ≤50 mL/min for women); adequacy of a subjective criterion for establishing the end point; RER_max_ ≥ 1.15; HR_max_ within 10 bpm of APMHR	CYC	CSI I	Different day	1 or 2 sub peak WRs, then 100% of the highest VO_2max_ reached from two CPET	≤5% difference between the ramp test and VP
				CSI II			
Niemeyer et al. [105]	30-s time average	< half of expected increase in VO_2_ (i.e. <4.5 mL·kg^-1^·min^-1^)	CYC	Ramp	10-min active	90% WR_peak_	CPET *vs*. VP: VO_2max_ difference ≤ 5%
Niemeyer et al. [106]	30-s time average	VO_2_ plateau (difference between modelled and actual value > 50% of the regression slope for the linear portion of the VO_2_-WR relationship)	CYC	Ramp	Different day	90% WR_peak_	CPET *vs*. VP: VO_2max_ difference ≤ 5%
Nolan et al. [107]	2×15-s	NS	TR	CSI	20-min passive	105% WR_peak_	CPET *vs*. VP: VO_2max_ difference ≤ 3%
						115% WR_peak_	
					60-min passive	105% WR_peak_	
						115% WR_peak_	
Poole et al. [37]	20 s	VO_2_ plateau of regarding the mL/min; RER_max_ ≥ 1.10, 1.15; HR_max_ within 10 bpm of APMHR; La_max_ ≥ 8 mmol	CYC	Ramp	Different day	105%WR_peak_ reached in the CPET	NS
Possamai et al. [108]	30-s intervals	plateau in VO_2_ and HR (i.e. ≤ 50 mL/min or ≤ 2 bpm) over the final two consecutive 30 s sampling periods; HR_max_ within 10 bpm of APMHR	CYC	CSI	15-min passive	5-min warm-up at the first stage of the CPET; 3-min of passive recovery; 2-min at 20 Watts; then increased 100% WR_peak_	CPET *vs*. VP: VO_2max_ difference ≤ 3%
Riboli et al. [109]	30-s intervals	VO_2_ plateau of 2.1 mL·kg^-1^·min^-1^	TR	CSI with 1 min stages	5-min passive	if the CPET did not show a VO_2_ plateau, a verification bout was performed as described by [38]; however, the intensity was not stated (i.e. 95 or 105%WR_peak_ reached in the CPET)	NS
				CSI with 2 min stages			
				DisCSI			
Rossiter et al. [38]	15-s average	VO_2_ plateau (linear least squares fitting technique)	CYC	Ramp	5-min active	105%WR_peak_ reached in the CPET	NS
						95%WR_peak_ reached in the CPET	
Sabino-Carvalho et al. [110]	20-s average	NS	TR	DisCSI	3-min passive (standing on treadmill) and 7-min active (walking at 5 km/h)	2-min at 60% WR_peak_ and then ↑ 0.5 km/h > CPET-Speed_peak_	CPET *vs*. VP: VO_2max_ difference ≤ 2%
Scharhag-Rosenberger et al. [111]	3×10-s average	VO_2_ plateau (increase < than one-third of the oxygen requirement of a stage change ~ 150 mL/min); RER_max_ ≥ 1.10; ± 10 bpm APMHR; La_max_ > 8 mmol	TR	DisCSI	10-min passive (VerifDay1)	1 min at 60% CPET-Speed_peak_ and then continued at 110% (or 115% if necessary, a second VF bout in VerifDay1) CPET-Speed_peak_	CPET *vs*. VP: VO_2max_ difference ≤ 5.5%
					Different day (VerifDay2)		
Scheadler and Devor [112]	30-s	NS	TR	CSI	Different day	8% slope/ individualized speed for a WR greater than CPET (mean estimated 10.2% WR_peak_)	CPET *vs*. VP: VO_2max_ difference ≤ 50 mL/min
Sedgeman et al. [113]	15-s time average	VO_2_ plateau of 2.1 mL·kg^-1^·min^-1^ during the last two 15-s average samples	CYC	CSI	3-min active	WR_peak_ minus 2-stages	CPET *vs*. VP: VO_2max_ difference ≤ 3%
						105%WR_peak_	
Stachenfeld et al. [114]	20-s averaging	VO_2_ plateau of 150 mL/min; RER_max_ ≥ 1.10, 1.15; ≥ 85% APMHR; La_max_ ≥ 8 mmol	CYC	CSI	Different day	115% WR_peak_ reached in the CPET or 125% if the plateau has not been attained	VO_2_ plateau of 150 mL/min
Straub et al. [115]	15-s time average	NS	CYC	Ramp	10-min passive	1^st^ min: 60% WR_peak_ and then 110% WR_peak_	NS
Strom et al. [116]	30-s time average	NS	TR	CSI	3-min active (walking pace of 67 m/min)	2 stages < CPET-WR_peak_	CPET *vs*. VP: VO_2max_ difference ≤ 3%
Taylor et al. [117]	15-breath average	NS	TR	CSI	15-min active or passive	1^st^ min at 10 km/h (5% slope) and then ↑ 1 km/h > CPET-Speed_peak_	NS
Tucker et al. [118]	2×15-s	NS	CYC	CSI	5–10 min active	100%WR_peak_	NS
Vogiatzis et al. [119]	NS	NS	CYC	CSI	20-min passive	110% WR_peak_	NS
Weatherwax et al. [120]	2×15-s	NS	TR	Pseudo-ramp protocol	20-min passive	105% WR_peak_ (Trials 1 and 2)	CPET *vs*. VP: VO_2max_ difference ≤ 3%
Weatherwax et al. [15]	2×15-s	NS	TR	Pseudo-ramp protocol	20-min passive	105% WR_peak_	CPET *vs*. VP: VO_2max_ difference ≤ 3%
Weatherwax et al. [121]	2×15-s	NS	TR	Pseudo-ramp protocol	20-min passive	105% WR_peak_	CPET *vs*. VP: VO_2max_ difference ≤ 3%
Weatherwax et al. [122]	2×15-s	NS	TR	DisCSI	20-min passive	3 min at 4.82 km/h and then ↑ 0.64 km/h > CPET-Speed_peak_ (males)	CPET *vs*. VP: VO_2max_ difference ≤ 3%
						3 min at 4.82 km/h and then ↑ 0.48 km/h > CPET-Speed_peak_ (females)	
Wilhelm et al. [123]	10-s moving average	NS	CYC	CSI	5-min passive	105%WR_peak_	NS
Williams et al. [124]	20-s	NS	CYC	Ramp	5-min active	105%WR_peak_	NS
Wingo et al. [125]	2×30-s	VO_2_ plateau of 135 mL/min; HR within 5 bpm of that on the control test was obtained	CYC	CSI control	20-min passive	100% WR_peak_ (if <1-min was completed during the last stage of the CPET) or 25 Watts > CPET-WR_peak_ (if ≥1-min was completed during the last stage of the CPET)	VO_2_ plateau of 135 mL/min
				CSI post-15 min			
				CSI post-45 min			
Yeh et al. [126]	NS	NS	TR	CSI	10-min passive	1 km/h > CPET-Speed_peak_ or 5% slope every minute until exhaustion	NS

**Abbreviations:** *APMHR* = age-predicted maximal heart rate; *CPET* = cardiopulmonary exercise test; *CSI* = continuous step-incremented; *CV* = coefficient of variation; *CYC* = cycling; *DisCSI* = discontinuous step-incremented; *HR* = heart rate; *HR*_*max*_ = maximal heart rate; *La*_*max*_ = maximal blood lactate concentration; *NS* = not stated; *RER*_*max*_ = maximal respiratory exchange ratio; *RPE* = rating of perceived exertion; *SD* = standard deviation; *SPV* = self-paced maximal oxygen uptake; *TR* = treadmill; *VO*_*2*_ = oxygen uptake; *VO*_*2max*_ = maximal oxygen uptake; *VP* = verification phase; *WR* = work rate; *WR*_*peak*_ = peak work rate. *Note*: whenever possible, authors were contacted to provide unpublished data.

In terms of processing respiratory VO_2_ data at volitional exhaustion, the most common approach was based on time averages. Thirty-eight studies (48%) reported stationary time averages of 5- to 30-s, whereas 29 (36%) used VO_2_ data points at fixed intervals of 15- to 30-s, two studies (3%) used 15-breath averages, two studies (3%) used 10-25-s moving averages, one (1%) used 10-s epochs, two (3%) used 20-s rolling averages, one (1%) used 30-s rolling means, and one study (1%) used Douglas bag collections. Four studies (5%) did not detail which VO_2_ data processing method was applied.

Regarding the period between the CPET and verification phase procedure, 34 studies (43%) used a short-term active recovery (e.g. pedaling at light-intensity, walking at a slow pace, or stretching) of 1, 3, 5, 6, 8, 10, or 5–10 min, while 26 studies (33%) employed passive recovery of 5, 6, 9, 10, 15, 20, 60, or 60–90 min. Two studies (3%) employed a combination of passive and active recovery and another (1%) used a self-paced approach where participants were permitted to choose their own WR. Three studies (4%) employed short-term recovery (e.g. 8–10 min) without stating whether it was active or passive. Fifteen studies (19%) carried out the verification phase on a different day to the CPET.

Sixty studies (75%) used square-wave verification phase protocols, while 20 studies (25%) used multistage verification protocols characterized by an initial warm-up stage. Overall, 53 studies (66%) adopted “supra WR_peak_” verification phases based upon the WR_peak_ achieved during the CPET (e.g. one treadmill or cycle ergometer WR stage higher than that completed in the CPET, or 105–130% of the WR_peak_ achieved in the previous CPET). Seven studies (9%) used only 100% of WR_peak_, while two other studies (3%) used both WR_peak_ and supra WR_peak_ verification phases. Three studies (4%) examined both sub and supra WR_peak_ within the same study and one study (1%) used a predicted WR based on the following formula to elicit the participant’s limit of tolerance within 180 s: power output = (finite work capacity ÷ 180 s) + critical power. Fourteen studies (18%) used only sub WR_peak_ verification phases ranging from 85%-95% WR_peak_ (typically two stages below the WR_peak_ achieved during the CPET) (see Table 2).

Forty-two studies (53%) employed cut-off points to analyze differences between the highest VO_2_ values obtained during the CPET and verification phase to confirm that VO_2max_ was likely attained. Criteria for VO_2max_ verification were frequently based on the intra-subject coefficient of variation acquired from the researchers’ laboratories or from published literature, including a VO_2_ difference ≤ 2%, ≤ 3%, ≤ 5.0–5.5%, ≤ 1.5–2.2 mL·kg^-1^·min^-1^, ≤ 50–150 mL/min, or alternative methods.

### Quantitative data synthesis: Differences between the highest VO_2_ attained in the CPET and verification phase

**Fig 2:**
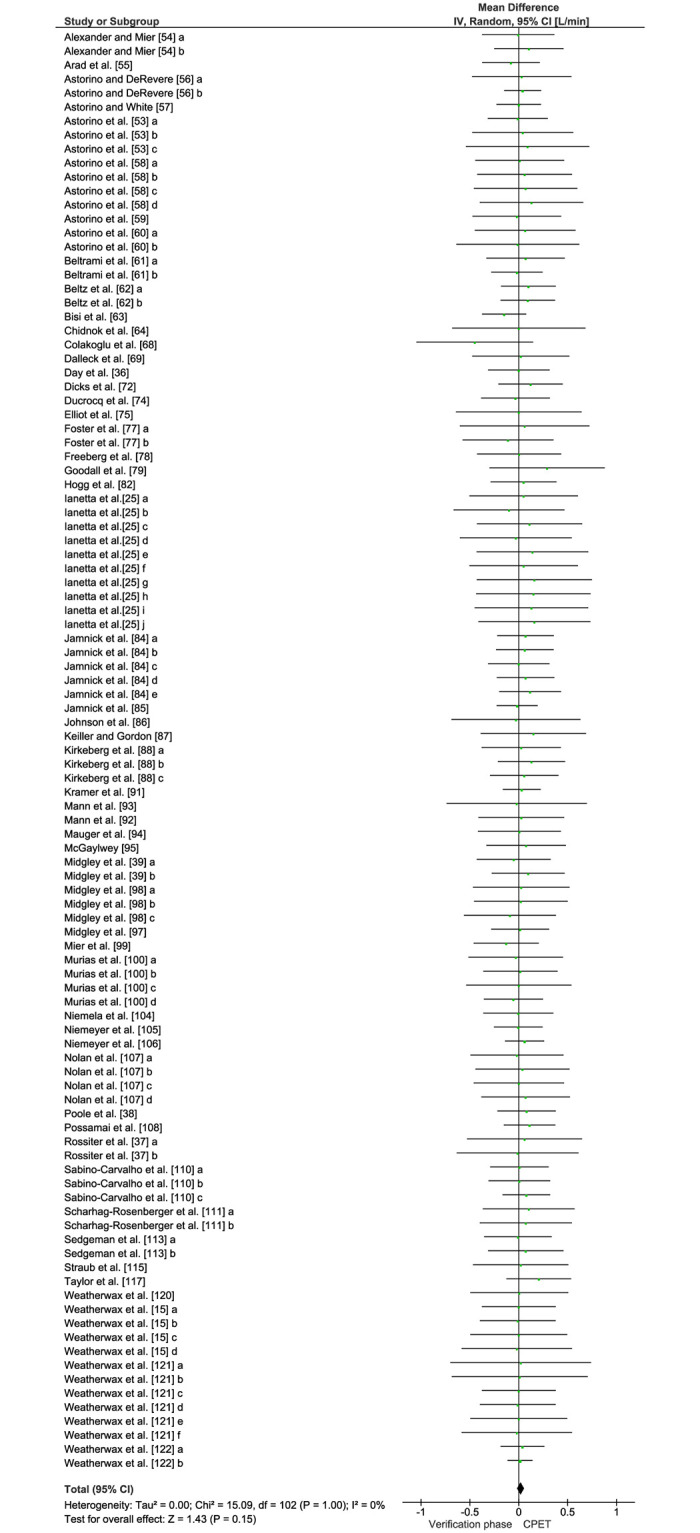
Forest plot of all studies included in the meta-analysis (k = 54) for the highest VO_2_ responses attained in the cardiopulmonary exercise test and verification phase using random effects analyses. Data are reported as mean differences (MD) adjusted for control data (95% CIs).

**Fig 3:**
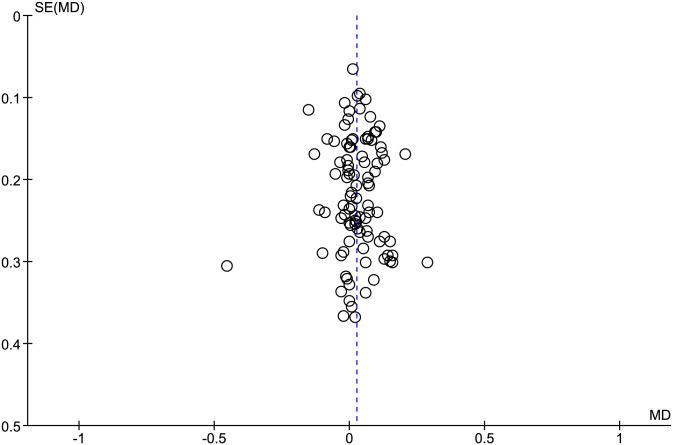
Funnel plot assessment of publication bias for the studies investigating the highest VO_2_ responses attained in the cardiopulmonary exercise test and verification phase.

Table 3 shows comparisons between the highest VO_2_ values elicited in the CPET and verification phase for each study. Fig 2 displays the forest plots of effect sizes and 95% CIs for the highest VO_2_ values (54 studies) based on the random effects meta-analysis results. Notably, the mean highest VO_2_ values were similar between the CPET and verification phase (mean difference = 0.03 [95% CI = -0.01 to 0.06] L/min, *P* = 0.15). Pooled data for VO_2max_ following the CPET and verification phase showed no significant heterogeneity among the studies overall (see Fig 2). Except for one of the included studies judged to have a high risk of bias [68], the meta-analyzed studies were judged to have a low-risk of bias as shown by the funnel plot (Fig 3).

**Table 3:** Overall comparisons in the meta-analyzed studies for the highest VO_2_ values attained in the cardiopulmonary exercise test (CPET) and verification phase (VP) (k = 54).

Study	Specific Experimental Condition		CPET			VP			% Weight	Mean Difference
			Mean [L/min]	SD [L/min]	Total	Mean [L/min]	SD [L/min]	Total		IV, Random, 95%CI [L/min]
Alexander and Mier [54]	CPET protocol (CSI)		3.79	0.39	11	3.80	0.49	11	1.00%	-0.01 [-0.38, 0.36]
	CPET protocol (DisCSI)		3.94	0.40	11	3.84	0.45	11	1.00%	0.10 [-0.25, 0.46]
Arad et al. [55]	N/A		2.18	0.61	35	2.26	0.65	35	1.40%	-0.08 [-0.38, 0.22]
Astorino and DeRevere [56]	CPET-VP recovery (8 min) VP intensity (105% WR_peak_)		3.35	1.01	30	3.32	1.00	30	0.50%	0.03 [-0.48, 0.54]
	CPET-VP recovery (10 min) VP intensity (110% WR_peak_)		2.82	0.62	79	2.78	0.59	79	3.70%	0.04 [-0.15, 0.23]
Astorino and White [57]	N/A		3.00	0.45	30	3.00	0.45	30	2.50%	0.00 [-0.23, 0.23]
Astorino et al. [53]	Experimental groups (low CRF)		2.35	0.37	10	2.36	0.33	10	1.40%	-0.01 [-0.32, 0.30]
	Experimental groups (moderate CRF)		3.32	0.58	10	3.28	0.60	10	0.50%	0.04 [-0.48, 0.56]
	Experimental groups (high CRF)		4.38	0.70	10	4.29	0.74	10	0.30%	0.09 [-0.54, 0.72]
Astorino et al. [58]	Training effect (HIIT-Baseline)		2.51	0.62	14	2.50	0.61	14	0.60%	0.01 [-0.45, 0.47]
	Training effect (HIIT—Week 3)		2.66	0.67	14	2.60	0.64	14	0.60%	0.06 [-0.43, 0.55]
	Training effect (Control—Baseline)		2.94	0.72	14	2.87	0.71	14	0.50%	0.07 [-0.46, 0.60]
	Training effect (Control—Week 3)		2.97	0.74	14	2.84	0.69	14	0.50%	0.13 [-0.40, 0.66]
Astorino et al. [59]	N/A		2.55	0.62	14	2.57	0.61	14	0.60%	-0.02 [-0.47, 0.43]
Astorino et al. [60]	CPET-VP recovery (at least 24h)		2.37	0.69	15	2.31	0.75	15	0.50%	0.06 [-0.45, 0.58]
	CPET-VP recovery (60 to 90 min)		2.72	0.65	9	2.73	0.72	9	0.30%	-0.01 [-0.64, 0.62]
Beltrami et al. [61]	Experimental groups (control group)		4.50	0.58	13	4.43	0.46	13	0.80%	0.07 [-0.33, 0.47]
	Experimental groups (reverse group)		4.52	0.36	13	4.54	0.33	13	1.90%	-0.02 [-0.28, 0.24]
Beltz et al. [62]	CPET protocol (SPV)		3.84	0.28	16	3.74	0.50	16	1.70%	0.10 [-0.18, 0.38]
	CPET protocol (Ramp)		3.86	0.28	16	3.77	0.50	16	1.70%	0.09 [-0.19, 0.37]
Bisi et al. [63]	N/A		2.41	0.13	11	2.56	0.36	11	2.60%	-0.15 [-0.38, 0.08]
Chidnok et al. [64]	N/A		4.32	0.61	7	4.32	0.69	7	0.30%	0.00 [-0.68, 0.68]
Colakoglu et al. [68]	N/A		4.11	0.69	9	4.56	0.60	9	0.40%	-0.45 [-1.05, 0.15]
Dalleck et al. [69]	N/A		2.33	0.76	18	2.31	0.76	18	0.50%	0.02 [-0.48, 0.52]
Day et al. [35]	N/A		3.64	0.70	38	3.64	0.70	38	1.30%	0.00 [-0.31, 0.31]
Dicks et al. [72]	N/A		3.84	0.65	28	3.72	0.60	28	1.20%	0.12 [-0.21, 0.45]
Ducrocq et al. [74]	N/A		3.73	0.47	13	3.76	0.45	13	1.10%	-0.03 [-0.39, 0.32]
Elliott et al. [75]	N/A		4.26	0.61	8	4.26	0.70	8	0.30%	0.00 [-0.64, 0.64]
Foster et al. [77]	VP exercise modality (TR)		4.09	0.97	20	4.03	1.16	20	0.30%	0.06 [-0.60, 0.72]
	VP exercise modality (CYC)		3.95	0.75	20	4.06	0.75	20	0.60%	-0.11 [-0.57, 0.35]
Freeberg et al. [78]	N/A		3.49	0.85	30	3.49	0.85	30	0.70%	0.00 [-0.43, 0.43]
Goodall et al. [79]	N/A		4.11	0.56	9	3.82	0.71	9	0.40%	0.29 [-0.30, 0.88]
Hogg et al. [82]	N/A		4.87	0.43	14	4.82	0.48	14	1.20%	0.05 [-0.29, 0.39]
Iannetta et al. [25]	WR_peak_ 5 W/min	1^st^ VP at 110% WR_peak_ (25 W/min)	3.35	0.68	11	3.30	0.65	11	0.4%	0.05 [-0.51, 0.61]
		2^nd^ VP at 110% WR_peak_ (5 W/min)	3.35	0.68	11	3.45	0.68	11	0.4%	-0.10 [-0.67, 0.47]
	WR_peak_ 10 W/min	1^st^ VP at 110% WR_peak_ (25 W/min)	3.44	0.67	11	3.33	0.62	11	0.4%	0.11 [-0.43, 0.65]
		2^nd^ VP at 110% WR_peak_ (10 W/min)	3.44	0.67	11	3.47	0.7	11	0.4%	-0.03 [-0.60, 0.54]
	WR_peak_ 15 W/min	1^st^ VP at 110% WR_peak_ (25 W/min)	3.44	0.69	11	3.3	0.68	11	0.4%	0.14 [-0.43, 0.71]
		2^nd^ VP at 110% WR_peak_ (15 W/min)	3.44	0.69	11	3.39	0.64	11	0.4%	0.05 [-0.51, 0.61]
	WR_peak_ 25 W/min	1^st^ VP at 110% WR_peak_ (25 W/min)	3.44	0.74	11	3.28	0.67	11	0.4%	0.16 [-0.43, 0.75]
		2^nd^ VP at 110% WR_peak_ (25 W/min)	3.44	0.74	11	3.29	0.66	11	0.4%	0.15 [-0.44, 0.74]
	WR_peak_ 30 W/min	1^st^ VP at 110% WR_peak_ (25 W/min)	3.44	0.72	11	3.31	0.67	11	0.4%	0.13 [-0.45, 0.71]
		2^nd^ VP at 110% WR_peak_ (30 W/min)	3.44	0.72	11	3.28	0.65	11	0.4%	0.16 [-0.41, 0.73]
Jamnick et al. [84]	CPET protocol (CSI_1_: 1-min stage length)		4.72	0.41	17	4.65	0.45	17	1.60%	0.07 [-0.22, 0.36]
	CPET protocol (CSI_3_: 3-min stage length)		4.62	0.42	17	4.56	0.46	17	1.50%	0.06 [-0.23, 0.36]
	CPET protocol (CSI_5_: 5-min stage length)		4.55	0.46	17	4.55	0.47	17	1.30%	0.00 [-0.31, 0.31]
	CPET protocol (CSI_7_: 7-min stage length)		4.44	0.42	17	4.37	0.46	17	1.50%	0.07 [-0.22, 0.36]
	CPET protocol (CSI_10_: 10-min stage length)		4.35	0.43	17	4.23	0.51	17	1.30%	0.12 [-0.20, 0.43]
Jamnick et al. [85]	N/A		3.24	0.57	57	3.25	0.57	57	3.00%	-0.02 [-0.23, 0.19]
Johnson et al. [86]	N/A		3.31	0.76	11	3.34	0.82	11	0.30%	-0.03 [-0.69, 0.63]
Keiller and Gordon [87]	N/A		3.65	0.71	11	3.50	0.58	11	0.50%	0.15 [-0.39, 0.69]
Kirkeberg et al. [88]	CPET protocol (short-term CSI)		4.43	0.48	12	4.41	0.54	12	0.80%	0.03 [-0.38, 0.43]
	CPET protocol (middle-term CSI)		4.40	0.46	12	4.27	0.40	12	1.00%	0.13 [-0.21, 0.47]
	CPET protocol (large-term CSI)		4.42	0.42	12	4.36	0.45	12	1.00%	0.06 [-0.29, 0.41]
Kramer et al. [91]	N/A		3.45	0.29	15	3.42	0.25	15	3.50%	0.03 [-0.16, 0.22]
Mann et al. [93]	N/A		4.11	0.78	10	4.13	0.85	10	0.30%	-0.02 [-0.74, 0.70]
Mann et al. [92]	N/A		3.80	0.87	32	3.78	0.92	32	0.70%	0.03 [-0.41, 0.46]
Mauger et al. [94]	N/A		4.66	0.55	14	4.65	0.59	14	0.70%	0.01 [-0.42, 0.43]
McGawley [95]	N/A		4.08	0.47	10	4.01	0.46	10	0.80%	0.08 [-0.33, 0.48]
Midgley et al. [39]	VP exercise modality (CYC)		3.86	0.39	10	3.92	0.47	10	0.90%	-0.05 [-0.43, 0.33]
	VP exercise modality (TR)		4.05	0.47	10	3.96	0.38	10	0.90%	0.10 [-0.28, 0.47]
Midgley et al. [98]	CPET protocol (CSI 1-min stages)		4.09	0.54	9	4.07	0.53	9	0.50%	0.03 [-0.47, 0.52]
	CPET protocol (DisCSI 2-min stages)		4.10	0.52	9	4.08	0.52	9	0.60%	0.02 [-0.46, 0.50]
	CPET protocol (DisCSI 3-min stages)		3.98	0.49	9	4.07	0.53	9	0.60%	-0.09 [-0.56, 0.38]
Midgley et al. [97]	N/A		4.03	0.42	16	4.01	0.44	16	1.50%	0.01 [-0.28, 0.31]
Mier et al. [99]	N/A		3.64	0.38	10	3.77	0.38	10	1.20%	-0.13 [-0.46, 0.20]
Murias et al. [100]	VP intensity (younger: 85% WR_peak_)		3.73	0.51	8	3.76	0.48	8	0.60%	-0.03 [-0.52, 0.45]
	VP intensity (younger: 105% WR_peak_)		3.90	0.65	22	3.89	0.64	22	0.90%	0.02 [-0.36, 0.40]
	VP intensity (older: 85% WR_peak_)		2.18	0.55	8	2.18	0.55	8	0.50%	0.00 [-0.54, 0.54]
	VP intensity (older: 105% WR_peak_)		2.52	0.54	23	2.57	0.51	23	1.40%	-0.05 [-0.36, 0.25]
Niemela et al. [104]	N/A		3.05	0.55	16	3.05	0.49	16	1.00%	0.00 [-0.36, 0.35]
Niemeyer et al. [105]	N/A		4.06	0.43	24	4.06	0.46	24	2.10%	0.00 [-0.25, 0.24]
Niemeyer et al. [106]	N/A		4.01	0.47	46	3.95	0.51	46	3.30%	0.06 [-0.14, 0.26]
Nolan et al. [107]	CPET-VP recovery (20 min) VP intensity (105% WR_peak_)		3.64	0.61	12	3.66	0.58	12	0.60%	-0.02 [-0.50, 0.46]
	CPET-VP recovery (20 min) VP intensity (115% WR_peak_)		3.68	0.59	12	3.64	0.61	12	0.60%	0.04 [-0.44, 0.52]
	CPET-VP recovery (60 min) VP intensity (105% WR_peak_)		3.60	0.58	12	3.60	0.58	12	0.60%	0.00 [-0.46, 0.46]
	CPET-VP recovery (60 min) VP intensity (115% WR_peak_)		3.65	0.54	12	3.58	0.60	12	0.60%	0.07 [-0.38, 0.52]
Poole et al. [37]	N/A		4.03	0.28	7	3.95	0.29	7	1.50%	0.08 [-0.22, 0.38]
Possamai et al. [108]	N/A		3.83	0.41	19	3.72	0.42	19	1.90%	0.11 [-0.15, 0.37]
Rossiter et al. [38]	VP intensity (105%WR_peak_)		4.15	0.50	5	4.09	0.45	5	0.40%	0.06 [-0.53, 0.65]
	VP intensity (95%WR_peak_)		4.11	0.48	5	4.12	0.53	5	0.30%	-0.01 [-0.64, 0.61]
Sabino-Carvalho et al. [110]	Pre-CPET intervention (IPC)		4.24	0.46	16	4.23	0.40	16	1.50%	0.01 [-0.29, 0.31]
	Pre-CPET intervention (Sham)		4.23	0.48	16	4.23	0.43	16	1.30%	0.01 [-0.31, 0.32]
	Pre-CPET intervention (Control)		4.23	0.38	16	4.15	0.32	16	2.20%	0.08 [-0.17, 0.32]
Scharhag-Rosenberger et al. [111]	CPET-VP recovery (same day after 10 min)		3.82	0.99	34	3.72	0.99	34	0.60%	0.10 [-0.37, 0.57]
	CPET-VP recovery (different day)		3.82	0.99	34	3.75	1.00	34	0.60%	0.07 [-0.40, 0.54]
Sedgeman et al. [113]	VP intensity (WR_peak_ minus 2-stages)		3.69	0.41	13	3.70	0.49	13	1.10%	-0.01 [-0.36, 0.34]
	VP intensity (105%WR_peak_)		3.71	0.51	13	3.64	0.50	13	0.90%	0.07 [-0.31, 0.46]
Straub et al. [115]	N/A		3.86	0.73	16	3.84	0.68	16	0.60%	0.02 [-0.47, 0.51]
Taylor et al. [117]	N/A		4.03	0.53	19	3.83	0.52	19	1.20%	0.21 [-0.13, 0.54]
Weatherwax et al. [120]	N/A		2.29	0.73	16	2.29	0.73	16	0.50%	0.00 [-0.50, 0.51]
Weatherwax et al. [15]	Training effect (standardized—baseline)		2.03	0.62	20	2.03	0.60	20	0.90%	0.00 [-0.38, 0.38]
	Training effect (standardized—week 12)		2.17	0.62	20	2.18	0.63	20	0.90%	-0.01 [-0.40, 0.38]
	Training effect (individualized—baseline)		2.37	0.79	19	2.37	0.77	19	0.50%	0.00 [-0.50, 0.50]
	Training effect (individualized—week 12)		2.63	0.89	19	2.65	0.89	19	0.40%	-0.02 [-0.59, 0.55]
Weatherwax et al. [121]	Training effect (control—baseline)		2.18	0.74	8	2.16	0.73	8	0.30%	0.02 [-0.70, 0.74]
	Training effect (control—week 12)		2.11	0.73	8	2.10	0.69	8	0.30%	0.01 [-0.69, 0.71]
	Training effect (standardized—baseline)		2.03	0.62	20	2.03	0.60	20	0.90%	0.00 [-0.38, 0.38]
	Training effect (standardized—week 12)		2.17	0.62	20	2.18	0.63	20	0.90%	-0.01 [-0.40, 0.38]
	Training effect (individualized—baseline)		2.37	0.79	19	2.37	0.77	19	0.50%	0.00 [-0.50, 0.50]
	Training effect (individualized—week 12)		2.63	0.89	19	2.65	0.89	19	0.40%	-0.02 [-0.59, 0.55]
Weatherwax et al. [122]	Experimental groups (males)		3.98	0.36	18	3.94	0.32	18	2.60%	0.04 [-0.19, 0.26]
	Experimental groups (females)		2.68	0.13	6	2.67	0.10	6	8.00%	0.01 [-0.12, 0.14]

**Abbreviations:** *CI* = confidence interval; *CPET* = cardiopulmonary exercise test; *CRF* = cardiorespiratory fitness level; *CSI* = continuous step-incremented; *CYC* = cycling; *DisCSI* = discontinuous step-incremented; *HIIT* = high-intensity interval training; *IPC* = ischemic preconditioning; *N/A* = not applicable; *TR* = treadmill; *SD* = standard deviation; *SPV* = self-paced maximal oxygen uptake; *VO*_*2*_ = oxygen uptake; *VP* = verification phase; *WR*_*peak*_ = peak work rate; W/min = incremental phase based on watts *per* minute. *Note*: whenever possible, authors were contacted to provide unpublished data. %Weight = weight attributed to each study due to its statistical power.

**Fig 4:**
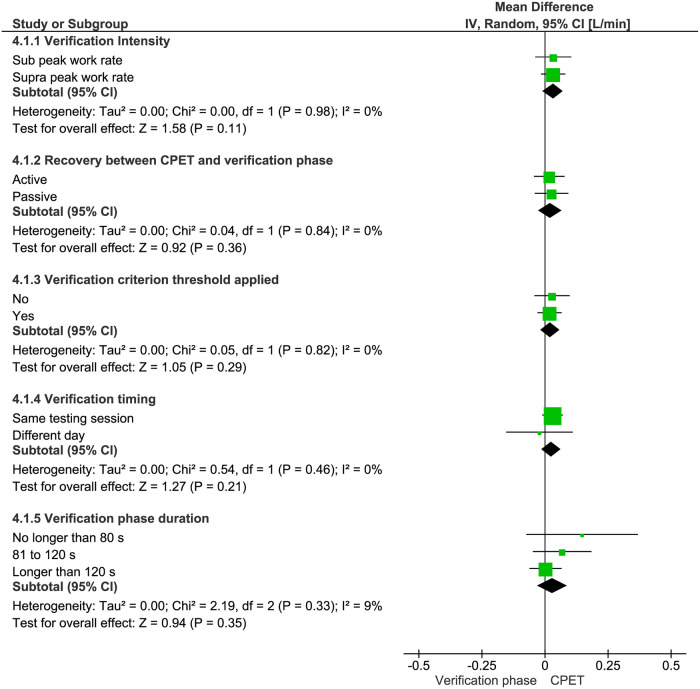
Mean differences (95% CIs) between the highest VO_2_ responses in the cardiopulmonary exercise test (CPET) and verification phase according to the verification-phase characteristics for intensity (i.e. sub WR_peak_ *vs*. supra WR_peak_), recovery (i.e. active *vs*. passive), adoption of criterion threshold (i.e. yes *vs*. no), timing (performed on the same day *vs*. a different day to the CPET), and duration (i.e. no longer than 80 s, from 81 to 120 s and longer than 120 s).

Results of subgroup analyses according to the characteristics of the verification phase protocol are summarized in Fig 4. There were no significant differences between the CPET and verification phase for the highest VO_2_ values attained after stratifying studies for verification-phase intensity (mean difference = 0.03 [95% CI = -0.01 to 0.07] L/min, *P* = 0.11), type of recovery utilized (mean difference = 0.02 [95%CI = -0.02 to 0.07] L/min, *P* = 0.36), VO_2max_ verification criterion adoption (mean difference = 0.02 [95% CI = -0.02 to 0.06] L/min, *P* = 0.29), verification procedure with regards to whether or not it was performed on the same day as the CPET (mean difference = 0.03 [95%CI -0.01 to 0.06] L/min, *P* = 0.21), or verification-phase duration (i.e. no longer than 80 s, from 81 to 120 s and longer than 120 s) (mean difference = 0.03 [95%CI -0.03 to 0.09] L/min, *P* = 0.35).

Subgroup analyses regarding sex, cardiorespiratory fitness level, exercise modality, and CPET protocol are summarized in Table 4. The median time to exhaustion was 665 s (IQR, 600 s) for the CPET and 148 s (IQR, 110 s) for the verification phase. Considering all sub-analyses presented in Table 4, there were no significant differences between the CPET and verification phase for VO_2max_ (*P* = 0.18 to *P* = 0.71).

**Table 4:** Subgroup analyses for the cardiopulmonary exercise test (CPET) and verification phase (VP).

	Time to exhaustion (s)			VO_2max_ (L/min)				
	N	CPET Mean ± SD	VP Mean ± SD	N	CPET Mean ± SD	VP Mean ± SD	Effect Size (95% CI)	*P*-value
**Sex**								
Male	146	734 ± 90	244 ± 43	630	3.95 ± 0.48	3.93 ± 0.50	0.02 (-0.02 to 0.08)	0.25
Female	23	659 ± 119	152 ± 46	68	2.63 ± 0.39	2.58 ± 0.40	0.05 (-0.08 to 0.12)	0.71
Both	677	765 ± 140	146 ± 28	941	3.24 ± 0.67	3.21 ± 0.67	0.03 (-0.04 to 0.08)	0.50
**Cardiorespiratory fitness level**								
Low	170	617 ± 111	150 ± 36	322	2.30 ± 0.65	2.32 ± 0.65	0.02 (-0.07 to 0.11)	0.63
Moderate	362	790 ± 101	200 ± 40	565	3.49 ± 0.61	3.45 ± 0.63	0.04 (-0.02 to 0.11)	0.21
High	346	792 ± 149	161 ± 27	716	3.94 ± 0.55	3.90 ± 0.55	0.04 (-0.02 to 0.08)	0.18
**Exercise modality**								
CYC	477	823 ± 143	155 ± 29	916	3.47 ± 0.59	3.45 ± 0.59	0.02 (-0.03 to 0.07)	0.43
TR	386	688 ± 110	189 ± 34	771	3.59 ± 0.58	3.56 ± 0.58	0.03 (-0.02 to 0.08)	0.22
**CPET protocol**								
DisCSI	92	876 ± 120	156 ± 28	169	3.90 ± 0.52	3.87 ± 0.51	0.03 (-0.05 to 0.11)	0.49
CSI	472	696 ± 105	209 ± 40	924	3.71 ± 0.56	3.69 ± 0.58	0.02 (-0.03 to 0.07)	0.38
Ramp	284	848 ± 171	121 ± 23	578	3.16 ± 0.63	3.13 ± 0.62	0.03 (-0.04 to 0.09)	0.44

Group weighted mean differences in maximal oxygen uptake (VO_2max_) according to sex, cardiorespiratory fitness level, exercise testing modality, and CPET protocol.

**Abbreviations:** *CI* = confidence interval; *CPET* = cardiopulmonary exercise test; *CSI* = continuous step-incremented; CYC = cycling; *DisCSI* = discontinuous step-incremented; *TR* = treadmill; *SD* = standard deviation; *VP* = verification phase.

## Discussion

A growing number of studies have included the verification phase procedure to increase confidence that the highest possible VO_2_ has been elicited by apparently healthy adults during a CPET. To the best of our knowledge this is the first systematic review and meta-analysis of these studies, and evidences that 90% of which have been published since 2009. The major findings were: (a) in general, the verification phase protocols elicited similar highest VO_2_ values to those obtained in the preceding CPET protocols; and (b) concordance between the highest VO_2_ values in the CPETs and verification phases were not affected by sex, cardiorespiratory fitness level, exercise modality, CPET protocol, or verification phase protocol.

The present systematic review and meta-analysis shows that the highest mean VO_2_ values elicited by verification phase bouts were similar to those elicited in continuous ramp or pseudo-ramp CPET protocols in the majority of studies. In fact, the mean absolute difference of 0.03 L/min for the 54 studies included in the meta-analysis represents a relative difference of only 0.85% between the highest VO_2_ values attained in the CPET and verification phase. This is within the most commonly adopted measures of test variability of 2–3% [57, 97]. The present findings also provide evidence that the similarity between the highest VO_2_ values attained during the CPETs and verification phases are not affected by sex, cardiorespiratory fitness, exercise modality, CPET protocol design, or how the verification phase was performed (see Table 4 and Fig 4). This contrasts with traditional VO_2max_ criteria, which are test-protocol dependent and vary according to the individual’s physical characteristics [28, 29]. Day et al. [35], for example, observed that participants with lower cardiorespiratory fitness had a lower tendency to exhibit a deceleration in the VO_2_ response at the end of a CPET compared to those with higher cardiorespiratory fitness and, therefore, are less likely to exhibit a VO_2_ plateau.

Six of the 54 meta-analyzed studies reported significant mean differences between the highest VO_2_ values observed in the CPET and verification phase [25, 55, 56, 68, 87, 95]. Astorino and DeRevere [56], for example, observed significantly higher mean VO_2max_ values by 0.03 and 0.04 L/min during the CPET than in the verification phase for two samples of participants heterogeneous for cardiorespiratory fitness. However, sub-group analyses revealed that while maximal VO_2_ in the CPET was higher than that attained in the verification phase for participants with moderate and high cardiorespiratory fitness, the opposite was true for those with lower cardiorespiratory fitness. Similar findings have been reported by Arad et al. [55], indicating that cardiorespiratory fitness level may be a key moderator of the differences between the highest VO_2_ values attained in the CPET and verification phase. A plausible explanation is that individuals with low cardiorespiratory fitness are more susceptible to stopping early during the CPET due to fatigue-associated symptoms [29], which would tend to result in lower VO_2_ values. In the present meta-analyses, the mean VO_2max_ in the verification phase was 8% higher than in the CPET in the low cardiorespiratory fitness group, but 12% and 10% higher in the CPET than in the verification phase in the moderate and high cardiorespiratory fitness groups, respectively (see Table 4). The lack of statistical significance, however, highlights the uncertainty regarding the effects of cardiorespiratory fitness on the differences between the highest VO_2_ values in the CPET and verification phase.

Regarding verification-phase duration, Keiller and Gordon [87] observed significantly higher VO_2_ values during the incremental treadmill CPETs versus the verification phase with a mean duration of approximately 2 min. This is consistent with the findings of McGawley [95] for 10 recreational runners who performed five consecutive treadmill CPET trials, plus an appended verification phase with a mean duration of < 2 min. Iannetta et al. [25] analyzed the VO_2_ responses to ramp-incremented cycling CPETs with WR increments of 5, 10, 15, 25, and 30 W/min, each followed by two verification phases performed at different WRs. The verification phase bouts performed at 110% of the WR_peak_ from ramp protocols with ramp rates of 25 and 30 W/min (i.e. short verification phase bouts of ~ 80 s) yielded VO_2_ values significantly lower than those attained in the CPETs. In contrast, the highest VO_2_ values attained during verification phase bouts based on slower WR increments of 5, 10, and 15 W/min, which allowed sufficient time for VO_2max_ attainment (i.e. 162, 122 and 103 s, respectively) were not different to those achieved in the preceding CPETs. Although the aforementioned studies suggest that verification phase duration is a key moderator for the mean differences between the highest VO_2_ observed in the CPET and verification phase, our sub-analysis found no difference for verification-phase durations of ≤ 80 s, ranging from 81 to 120 s, and > 120 s (see Fig 4). Notably, however, only three studies reported short durations of 80 s or less [25, 79, 113] and the lack of statistical significance may be due to the paucity of data.

In contrast to the aforementioned studies [25, 87, 95], Colakoglu et al. [68] observed significantly lower VO_2_ values in the CPET versus the verification phase in nine cycling and track and field athletes. According to Midgley et al. [97], if the mean highest VO_2_ attained in the verification phase is significantly higher than in the CPET, the investigator should consider that the CPET protocol was inadequate in eliciting the highest possible VO_2_ response in all or some of the participants. In the study by Colakoglu et al. [68], participants performed a prolonged step-incremented CPET consisting of one 4-min, three 2-min, and then 1-min increments until volitional exhaustion after 1 h of recovery from a submaximal CPET of at least four 5-min stages. It is feasible that the procedures performed before the maximal CPET may have led to poor participant motivation, lack of effort and premature fatigue in the following test. Additionally, the four verification phase bouts at 100%, 105%, 110%, and 115% of the WR_peak_ attained in the CPET were performed on four different days to the CPET without any preceding maximal exercise. This also may have positively favored the significantly higher mean VO_2_ values in the verification phase compared to the CPET and contrasts with the same-day verification phase used by Keiller and Gordon [87], McGawley [95], and Iannetta et al. [25].

An aim of the present systematic review was to suggest best practices for the application of verification phase protocols. The subgroup analyses revealed no systematic bias between the highest VO_2_ values observed in the CPET and verification phase according to the verification-phase intensity (i.e. sub WR_peak_ *vs*. supra WR_peak_), type of recovery between the CPET and verification phase (i.e. active *vs*. passive), whether a VO_2max_ criterion threshold was used for the CPET (i.e. yes *vs*. no), whether the verification phase was performed in the same testing session or on a different day, and the verification-phase duration (see Fig 4). Considering that differences in the verification phase procedure do not appear to influence its effectiveness, a specific verification procedure currently cannot be recommended. However, some caution must be exercised to avoid an inappropriately high verification-phase WR that results in a short test duration and insufficient time for the highest possible VO_2_ to be elicited [25], especially in untrained individuals characterized by slow VO_2_ kinetics [127]. Midgley et al. [97] stated that this is a plausible rationale for the early recommendations of Thoden [128], that individuals who do not reach 3 min in a supra WR_peak_ verification phase should undertake a subsequent verification phase at the same WR or one stage lower than verification-phase the last completed WR stage in the CPET. Poole and Jones [2] suggested that researchers should select a WR that is sufficiently higher than the WR_peak_ attained in the CPET, such as ~110% WR_peak_, to give the VO_2_ signal for the higher WR the opportunity to emerge from the extant noise. If the subsequent verification phase produces a VO_2_ plateau signifying VO_2max_, this signal would be lower than expected for the WR based on the previous VO_2_-WR slope. Conversely, Iannetta et al. [25] advocated a verification-phase WR lower than the WR_peak_ attained in the CPET in order to allow VO_2max_ to be elicited, since WRs above critical power should elicit VO_2max_ if the time to exhaustion is sufficiently long. Midgley et al. [39] proposed an alternative approach based on a multistage verification phase protocol that combines WRs below and above WR_peak_ to obtain a protocol that incorporates a supra WR_peak_ intensity with a relatively prolonged verification-phase duration. This approach has since been adopted in other studies [39, 53, 54, 61, 62, 64, 69, 76, 82, 87, 89, 90, 99, 104, 108, 110, 111, 115, 117, 122]. Notably, the only study to observe a statistically significant influence of verification phase intensity employed a multistage verification phase protocol incorporating 2 min at 50% of WR_peak_, increasing to 70% for an additional minute, and then 105 or 115% until volitional exhaustion [107]. Based on their findings, the authors recommended the use of 105% of the WR_peak_ attained in the CPET rather than 115% WR_peak_. The confounding results and various recommended approaches regarding the verification phase intensity indicates that more research is required before an evidence-based recommendation can be made.

Regarding the recovery time between the CPET and verification phase, intervals between 10–20 min have been commonly used, although in total a wide range of intervals from 1–3 min [65, 77, 88, 113] to 90 min [41] have been used. The present meta-analysis found no significant effect of recovery time on minimizing the difference between the mean VO_2_ elicited in the CPET and verification phase. An alternative method is to perform the verification phase on a separate day, although the additional visit to the laboratory and the day-to-day variability in VO_2max_ [129] might considerably reduce the utility and robustness of this approach. Scharhag-Rosenberger et al. [111] specifically investigated this issue by comparing a 10-min recovery to a verification phase performed on a separate day. No significant difference was observed between the two verification protocols, even though the time to exhaustion was significantly longer when the verification phase was performed on a separate day (2:06 ± 0:22 min *vs*. 2:42 ± 0:38 min). These findings suggest no advantage in performing the CPET and verification phase on separate days.

Inadequate data processing may negatively impact the utility of the verification phase procedure. Myers et al. [36] suggested small sampling intervals such as 5 and 10 s result in unacceptable variability in VO_2_ data, whereas large intervals such as 60 s may not be sufficiently sensitive to accurately track rapid changes in VO_2_ such as those observed in ramp and pseudo-ramp CPET protocols. Midgley et al. [130] observed that the reproducibility of VO_2max_ during continuous step-incremented treadmill CPETs is not affected by the length of the VO_2_ time-average interval between the range of 10 to 60 s, however, the actual VO_2max_ values were significantly different between time averages. The authors suggested that a 30-s stationary time-average for CPETs provides a good compromise between removing noise while maintaining the underlying trend in the VO_2_ data. However, no study to date has addressed the effect of the VO_2_ sampling interval on the verification phase.

A final issue to be addressed refers to appropriate criteria to accept that the highest possible VO_2_ has been achieved. The most common criterion used in the reviewed studies is that the highest VO_2_ observed in the verification phase should not exceed 3% of the highest VO_2_ obtained in the CPET. This threshold can be justified by the technical error of measurement and intra-individual biological variation associated with the determination of VO_2max_ [15, 56, 57, 62, 63, 69, 71, 78, 82, 86, 89–91, 95, 107, 108, 113, 116, 120–122]. The more restrictive value of ≤ 2% [97, 110] and the less restrictive values of ≤ 5–5.5% [104–106, 111] may also be appropriate for single or different-day variability. Further research is required before an appropriate verification-phase threshold can be recommended, which provides a high degree of confidence that the difference between the highest VO_2_ values observed in the CPET and verification phase are beyond the technical error of measurement and intra-individual biological variation.

Some limitations of the present review need to be acknowledged. First, the meta-analysis only included 79% of the participants that underwent CPET with verification phase protocols in the 80 studies included in the systematic review. This issue was due to unsuccessful attempts to acquire the required unpublished information from some authors. Second, the meta-analysis was based on comparison of the highest VO_2_ responses in the CPET and verification phase averaged across study participants. Noakes [131] criticized this approach, stating that the CPET is performed on individuals and not groups and, therefore, the group average approach does not identify individuals who may not have attained VO_2max_. A meta-analysis using individual participant data is therefore required. Finally, the present systematic review and meta-analysis comprised only apparently healthy adults and it is still unclear to what extent the use of the verification phase procedure is applicable to special or clinical populations. A growing number of studies have included special or clinical populations such as obese adults [132, 133], breast and prostate cancer survivors [134], wheelchair athletes [135], individuals with spinal-cord injuries [136], patients with heart failure [137] or cystic fibrosis [138–140], and pediatric populations [141–147], including children with spina bifida in an outpatient condition [148], and adolescents with cystic fibrosis [149].

## Conclusions

The present meta-analysis showed that the effect sizes calculated from the highest mean VO_2_ in apparently healthy adults were similar between CPETs and verification phases performed on a cycle ergometer or treadmill. Furthermore, mean differences between the highest VO_2_ values elicited in the CPETs and verification phases were not affected by participant characteristics, exercise modality, or the CPET and verification protocol design. Our findings indicate that from a practical perspective, different procedures may be applied to establish similar highest mean VO_2_ responses during the verification phase as compared to the ramp or continuous step-incremented CPETs. It is worth mentioning, however, that some caution must be exercised concerning the selection of sub or supra WR_peak_ verification phases, since any exercise above the critical power must be of sufficient duration to allow the achievement of the highest possible VO_2_ response in the verification phase. Our data reinforce the notion that a verification phase applied after ramp or continuous step-incremented CPETs may provide additional and unbiased evidence that the highest possible VO_2_ has been achieved. On the other hand, the invalidation of the highest VO_2_ obtained in CPETs by subsequent verification phases was less likely on a group basis. The mean differences in highest VO_2_ responses were typically within the test-retest variability of the experimental protocols employed. Accordingly, our findings support the usefulness of the verification phase to confirm the likely attainment of VO_2_ on incremental CPET. However, the necessity or mandatory application of the verification phase, especially constant supra WR_peak_ verification bouts, in all CPET situations remains open to question.

## Supporting information

S1 Checklist PRISMA 2009 checklist. (DOCX)

S1 Text Search strategy. (DOCX)
