# Supplementary material for: Is a verification phase useful for confirming maximal oxygen uptake in apparently healthy adults? A systematic review and meta-analysis
Source: PLoS One. 2021 Feb 17;16(2):e0247057. doi: 10.1371/journal.pone.0247057 (PMC7888616; doi:10.1371/journal.pone.0247057)
Supplement: S1 Text — (DOCX) [file pone.0247057.s002.docx]

**Supplementary appendix e-1: Search strategies for all databases**

1. Search Strategy for MEDLINE (Pubmed):

((("Exercise test"[Mesh] OR "Exercise test"OR "Exercise tests" OR "Test, Exercise" OR "Tests, Exercise" OR "Bicycle Ergometry Test" OR "Bicycle Ergometry Tests" OR "Test, Bicycle Ergometry" OR "Fitness Testing" OR "Testing, Fitness" OR "Step Test" OR "Step Tests" OR "Test, Step" OR "Step exercise test" OR "Stress Test" OR "Stress Tests" OR "Test, Stress" OR "Treadmill Test" OR "Test, Treadmill" OR "Treadmill Tests" OR "Physical Fitness Testing" OR "Cardiopulmonary Exercise Test" OR "Cardiopulmonary Exercise Tests" OR "Exercise Test, Cardiopulmonary" OR "Test, Cardiopulmonary Exercise" OR "incremental test" OR "incremental tests" OR "incremental exercise" OR "Graded Exercise Test" OR "Graded Exercise Tests" OR "Graded Exercise Testing" OR "Graded-Exercise test" OR "GXT" OR "Graded maximal exercise tests" OR "Graded maximal exercise test" OR "Maximal oxygen uptake test" OR "Maximal oxygen uptake tests" OR "maximal oxygen uptake" OR "maximal oxygen consumption" OR "maximum oxygen consumption" OR "maximal oxygen" OR "VO2" OR "VO2 uptake" OR "VO2 consumption" OR "VO2max" OR "VO2 max" OR "VO2MAX" OR "VO2 peak" OR "VO2peak" OR "peak oxygen uptake" OR "VO2 plateau") AND ("Verification" OR "Verification bout" OR "Verification phase" OR "Verification criteria" OR "Verification Stage" OR "Verification testing" OR "Verification test" OR "Supramaximal verification" OR "linear phase" OR "supramaximal test" OR "supramaximal testing" OR "GXT-verification" OR "Maximal verification oxygen uptake" OR "exhaustive square-wave run" OR "Higher power output" OR "Speeds above VO2max")) AND ("Healthy Volunteers"[Mesh] OR "Healthy Volunteers" OR"Healthy Volunteer" OR "Healthy" OR "Healthy Participants" OR "Healthy Participant" OR "Healthy Subjects" OR "Healthy Subject" OR "Human Volunteers" OR "Human Volunteer" OR "Normal Volunteers" OR "Normal Volunteer" OR "Healthy Individuals" OR "Healthy Individual" OR "Runner" OR "Runners" OR "Active subjects" OR "Athlete" OR "Athletes" OR "Athletics" OR "Participant" OR "Participants" OR "Active participant" OR "Active participants" OR "Recreational-trained" OR "Men" OR "Women" OR "Adult" OR "Adults" OR "Young"))

2. Search Strategy for Cochrane Library

(“Verification" OR “Verification bout" OR “Verification phase” OR “Verification criteria” OR “Verification Stage” OR “Verification testing” OR “Verification test” OR “Supramaximal verification” OR "linear phase" OR “supramaximal test” OR “supramaximal testing” OR “GXT-verification” OR “Maximal verification oxygen uptake” OR “exhaustive square-wave run” OR “Higher power output” OR “Speeds above VO2max”):ti,ab,kw AND ("Healthy Volunteers” OR“Healthy Volunteer” OR “Healthy” OR “Healthy Participants” OR “Healthy Participant” OR “Healthy Subjects” OR “Healthy Subject” OR “Human Volunteers” OR “Human Volunteer” OR “Normal Volunteers” OR “Normal Volunteer” OR “Healthy Individuals” OR “Healthy Individual” OR “Runner” OR “Runners” OR “Active subjects” OR “Athlete” OR “Athletes” OR “Athletics” OR “Participant” OR “Participants” OR “Active participant” OR “Active participants” OR “Recreational-trained” OR “Men” OR “Women” OR “Adult” OR “Adults” OR “Young”):ti,ab,kw AND (“Exercise tests” OR “Test, Exercise” OR “Tests, Exercise” OR “Bicycle Ergometry Test” OR “Bicycle Ergometry Tests” OR “Test, Bicycle Ergometry” OR “Fitness Testing” OR “Testing, Fitness” OR “Step Test” OR “Step Tests” OR “Test, Step” OR "Step exercise test" OR “Stress Test” OR “Stress Tests” OR “Test, Stress” OR “Treadmill Test” OR “Test, Treadmill” OR “Treadmill Tests” OR “Physical Fitness Testing” OR “Cardiopulmonary Exercise Test” OR “Cardiopulmonary Exercise Tests” OR “Exercise Test, Cardiopulmonary” OR “Test, Cardiopulmonary Exercise” OR “incremental test” OR “incremental tests” OR "incremental exercise" OR "Graded Exercise Test" OR "Graded Exercise Tests" OR "Graded Exercise Testing” OR “Graded-Exercise test” OR “GXT” OR “Graded maximal exercise tests” OR “Graded maximal exercise test” OR “Maximal oxygen uptake test” OR “Maximal oxygen uptake tests” OR “maximal oxygen uptake” OR “maximal oxygen consumption” OR “maximum oxygen consumption” OR “maximal oxygen” OR “VO2” OR “VO2 uptake” OR “VO2 consumption” OR “VO2max” OR “VO2 max” OR “VO2MAX” OR “VO2 peak” OR “VO2peak” OR “peak oxygen uptake” OR “VO2 plateau”):ti,ab,kw"

3. Search Strategy for web of science

#1 TS=(“Exercise tests” OR “Test, Exercise” OR “Tests, Exercise” OR “Bicycle Ergometry Test” OR “Bicycle Ergometry Tests” OR “Test, Bicycle Ergometry” OR “Fitness Testing” OR “Testing, Fitness” OR “Step Test” OR “Step Tests” OR “Test, Step” OR "Step exercise test" OR “Stress Test” OR “Stress Tests” OR “Test, Stress” OR “Treadmill Test” OR “Test, Treadmill” OR “Treadmill Tests” OR “Physical Fitness Testing” OR “Cardiopulmonary Exercise Test” OR “Cardiopulmonary Exercise Tests” OR “Exercise Test, Cardiopulmonary” OR “Test, Cardiopulmonary Exercise” OR “incremental test” OR “incremental tests” OR "incremental exercise" OR "Graded Exercise Test" OR "Graded Exercise Tests" OR "Graded Exercise Testing” OR “Graded-Exercise test” OR “GXT” OR “Graded maximal exercise tests” OR “Graded maximal exercise test” OR “Maximal oxygen uptake test” OR “Maximal oxygen uptake tests” OR “maximal oxygen uptake” OR “maximal oxygen consumption” OR “maximum oxygen consumption” OR “maximal oxygen” OR “VO2” OR “VO2 uptake” OR “VO2 consumption” OR “VO2max” OR “VO2 max” OR “VO2MAX” OR “VO2 peak” OR “VO2peak” OR “peak oxygen uptake” OR “VO2 plateau”)

#2 TS=("Healthy Volunteers” OR“Healthy Volunteer” OR “Healthy” OR “Healthy Participants” OR “Healthy Participant” OR “Healthy Subjects” OR “Healthy Subject” OR “Human Volunteers” OR “Human Volunteer” OR “Normal Volunteers” OR “Normal Volunteer” OR “Healthy Individuals” OR “Healthy Individual” OR “Runner” OR “Runners” OR “Active subjects” OR “Athlete” OR “Athletes” OR “Athletics” OR “Participant” OR “Participants” OR “Active participant” OR “Active participants” OR “Recreational-trained” OR “Men” OR “Women” OR “Adult” OR “Adults” OR “Young”)

#3 TS=(“Verification" OR “Verification bout" OR “Verification phase” OR “Verification criteria” OR “Verification Stage” OR “Verification testing” OR “Verification test” OR “Supramaximal verification” OR "linear phase" OR “supramaximal test” OR “supramaximal testing” OR “GXT-verification” OR “Maximal verification oxygen uptake” OR “exhaustive square-wave run” OR “Higher power output” OR “Speeds above VO2max”)

#4 #1 AND #2 AND #3

4. Search Strategy for SPORTDiscus (Ebsco)

(“Exercise tests” OR “Test, Exercise” OR “Tests, Exercise” OR “Bicycle Ergometry Test” OR “Bicycle Ergometry Tests” OR “Test, Bicycle Ergometry” OR “Fitness Testing” OR “Testing, Fitness” OR “Step Test” OR “Step Tests” OR “Test, Step” OR "Step exercise test" OR “Stress Test” OR “Stress Tests” OR “Test, Stress” OR “Treadmill Test” OR “Test, Treadmill” OR “Treadmill Tests” OR “Physical Fitness Testing” OR “Cardiopulmonary Exercise Test” OR “Cardiopulmonary Exercise Tests” OR “Exercise Test, Cardiopulmonary” OR “Test, Cardiopulmonary Exercise” OR “incremental test” OR “incremental tests” OR "incremental exercise" OR "Graded Exercise Test" OR "Graded Exercise Tests" OR "Graded Exercise Testing” OR “Graded-Exercise test” OR “GXT” OR “Graded maximal exercise tests” OR “Graded maximal exercise test” OR “Maximal oxygen uptake test” OR “Maximal oxygen uptake tests” OR “maximal oxygen uptake” OR “maximal oxygen consumption” OR “maximum oxygen consumption” OR “maximal oxygen” OR “VO2” OR “VO2 uptake” OR “VO2 consumption” OR “VO2max” OR “VO2 max” OR “VO2MAX” OR “VO2 peak” OR “VO2peak” OR “peak oxygen uptake” OR “VO2 plateau” ) AND ( "Healthy Volunteers” OR“Healthy Volunteer” OR “Healthy” OR “Healthy Participants” OR “Healthy Participant” OR “Healthy Subjects” OR “Healthy Subject” OR “Human Volunteers” OR “Human Volunteer” OR “Normal Volunteers” OR “Normal Volunteer” OR “Healthy Individuals” OR “Healthy Individual” OR “Runner” OR “Runners” OR “Active subjects” OR “Athlete” OR “Athletes” OR “Athletics” OR “Participant” OR “Participants” OR “Active participant” OR “Active participants” OR “Recreational-trained” OR “Men” OR “Women” OR “Adult” OR “Adults” OR “Young” ) AND ( =(“Verification" OR “Verification bout" OR “Verification phase” OR “Verification criteria” OR “Verification Stage” OR “Verification testing” OR “Verification test” OR “Supramaximal verification” OR "linear phase" OR “supramaximal test” OR “supramaximal testing” OR “GXT-verification” OR “Maximal verification oxygen uptake” OR “exhaustive square-wave run” OR “Higher power output” OR “Speeds above VO2max”)
