# Supplementary material for: Is a verification phase useful for confirming maximal oxygen uptake in apparently healthy adults? A systematic review and meta-analysis
Source: PLoS One. 2021 Feb 17;16(2):e0247057. doi: 10.1371/journal.pone.0247057 (PMC7888616; doi:10.1371/journal.pone.0247057)
Supplement: Attachment — Submitted filename: Comments to the authors.pdf [file pone.0247057.s003.pdf]

This meta-analysis evaluated the validity of a verification phase to confirm the achievement of VO2max. Although the authors will notice that I am very critical of the model for reasons that I hope are clear and evident in my comments, I have to recognize that this is a very detailed analysis and that the data have the potential to make a meaningful contribution to the literature. However, I believe that the authors need to make some major adjustments in the structure of this manuscript so that the correct message is delivered. I believe that this could be solved relatively easily, but this would imply a shift in the interpretation of physiological bases for supporting the idea of the verification phase as a valid approach to satisfy the plateau criterion for achievement of VO2max. In fact, towards the end of the manuscript, I realized that the authors fully understand the limitations of the model. However, for reasons that are difficult for me to understand at this point, they still present the model as valid to test something that it cannot test. I have provided extensive (and admittedly repetitive) feedback on this aspect throughout my review. I apologize for this, but I was trying to be as clear as possible with my words so that the chances of misunderstanding my position are minimized. I truly hope that the authors are willing to make some important changes, as I consider the data very strong and I fully respect and value the amount of work put into this analysis.

Introduction

Lines 55-58: This sentence is too long and difficult to read as presented. For example, I do not think that the word “neuromuscular” is needed here. It is obvious that neuromuscular function is important for performance and it will impact vascular function to a given extent. However, VO2max is not defined by neuromuscular performance in my view. Regardless, the authors should shorten this sentence.

Line 59: It is interesting that the definition of VO2max included cardiovascular, pulmonary, neuromuscular, and metabolic responses, but the authors refer to the test as cardiopulmonary (CPET). I never understood this term, as it excludes the vascular component, which is a key component of O2 distribution. I do not think that I am asking for this to be changed, but I wanted to highlight that I do not like it.

Lines 70-72: It is surprising that the work of Iannetta et al. (Am J Physiol Regul Integr Comp Physiol. 2020 Jul 22. doi: 10.1152/ajpregu.00126.2020; J Appl Physiol. 2019 Dec 1;127(6):1519-1527) and Keir et al. (Appl Physiol Nutr Metab. 2018 Sep;43(9):882-892) is not mentioned here, as it highlights important aspects of ramp incremental tests that are often ignored and that have resulted in misinterpretations of: 1) the link between ramp incremental and constant-load VO2 and work rates; 2) the lack of validity in the idea of using a supra peak work rate intensity for the verification of the VO2max response (more on this will certainly arrive in later comments).

Lines 74: It remains surprising that the authors discuss that more needs to be learned about using information from ramp incremental tests and have not even mentioned the manuscripts indicated

above (I understand that one of them might be too recent, but I find the other omissions surprising).

Lines 75-76: This is true. For example, the supra peak work rate intensities that are often used represent a good example of what could be a wrong protocol. Additionally, if motivation is an issue, then the verification ride would not fix this problem!

Lines 77-104: I think the authors do a good job in this paragraph identifying important issues. However, this paragraph is a bit redundant and convoluted. This section could be shortened by ~30% to streamline the message.

Line 94: Second VO2max is missing the letter "x".

Lines 105-129: Well, there are several conceptual limitations in this section. First, I will start by saying that a wrong concept repeated by many people, remains a wrong concept (even when some of the people supporting the concept are dominant figures in the field). Let's start by saying that, by definition, there is no such thing as supramaximal. In the end, the work rate obtained at the end of a ramp incremental test is far from maximal. It is simply a peak value, that will be greater or smaller depending on the characteristics of the ramp. In fact, it has been shown that whereas VO2max (or peak if you prefer) remains constant across a wide range of slope during a ramp incremental test, the power output (PO) varies widely depending on the slope of the ramp. For example, Iannetta et al. (J Appl Physiol. 2019 Dec 1;127(6):1519-1527) showed that whereas steeper ramps elicit the greatest peakPOs, less steep ramps result in progressively lower peak POs. This demonstrates that the so-called "supramaximal" work rates are just an illusion. "Supramaximal" in relation to what? This has been further indicated in a study that showed that performing sub peak work rate exercise to exhaustion is more likely to result in the achievement of higher VO2 values than performing supra peak work rate exercise (Am J Physiol Regul Integr Comp Physiol. 2020 Jul 22. doi: 10.1152/ajpregu.00126.2020). Although this might sound counter intuitive to the naïve reader, it should be physiologically expected by anyone who understands how ramp incremental testing affects the development of the slow component of VO2. See Appl Physiol Nutr Metab. 2018 Sep;43(9):882-892 and Med Sci Sports Exerc. 2020 Mar 20. doi: 10.1249/MSS.0000000000002343 for more details.
Second, and based on this comment, I will add that: 1) the statement that "a continuous CPET followed by an appended supramaximal verification phase is conceptually similar to the discontinuous tests most commonly used from the 1920s to the 1970s, but with the notable advantage of requiring only a single visit to the laboratory" is simply wrong. I would highly recommend that the authors read the papers indicated above to fully appreciate the idea that there is no such thing as a "supramaximal" work rate; 2) it is surprising that experts in VO2 kinetics such as Poole and Jones have made the mistake or recommending that "supramaximal" intensities should be used for the verification rides. As indicated in the papers highlighted above, 110% of a typical ramp test (or even 1-min step) might not allow for enough time for the VO2 response to be fully expressed, thus resulting in VO2 values that are lower than those obtained

during the ramp incremental test, and creating the false idea that VO2max has been achieved when this cannot be demonstrated.

Lines 134-137: I would argue that a recent paper (Am J Physiol Regul Integr Comp Physiol. 2020 Jul 22. doi: 10.1152/ajpregu.00126.2020) has demonstrated that the verification phase is a flawed approach to confirm that VO2max has been achieved. In fact, this paper has indicated what type of verification ride would be most adequate in order to achieve the highest possible VO2 (which most likely represents VO2max, as derived from the ramp incremental test!)

Lines 137-139: Unfortunately, this meta-analysis cannot achieve this goal. All the authors can do is to provide information on what others have obtained from a ramp or step test and compare those values with the verification rides. Given some of the comments that I made before, the authors should understand that the idea of a “supramaximal” work rate to determine the plateau criterion is flawed. This is not something that is up to debate. It is just a fact based on published data. Then, given the limitation of the model and the inability to establish a plateau response as originally proposed, the verification ride is simply an extra ride that might make people feel more confident with the idea that the highest possible VO2 has been achieved. From a personal perspective, I have no issues with people doing this. However, the vast majority of the data indicate no significant differences between the highest VO2 from the ramp incremental test and the verification ride. When differences exist, those typically fall within the measurement error and on both sides of the probability (i.e., VO2 could be higher in either the ramp or the verification).

As a summary of this section, I think that this meta-analysis, as proposed, is set to fail as it cannot answer the question that it is proposed to answer. My recommendation would be that the authors simply try to answer whether there are differences in the highest VO2 values obtained from ramp or step tests compared to a verification ride. Even if differences existed, there is no way that the authors could claim that VO2max has been verified as the “supramaximal” intensities are rather arbitrary and often ignore the most basic physiological responses for VO2 adjustments. In my view, the introduction should describe the limitations with the idea that VO2max can be verified (i.e., the “supramaximal” concept is flawed and “submaximal” verification phases has been shown to work as well), while highlighting that there might still be value in trying to confirm that the highest possible VO2 was achieved, which can be done in a variety of ways (as demonstrated later in this study). However, I find it critical that the erroneous idea that VO2max is verified because a “supramaximal” intensity was added to the evaluation is not perpetuated.

Methods

General comment: Although I have participated in meta-analysis studies and reviewed some of them, I have to admit that statistical analysis is not my area of expertise. Thus, even though the information seems correct, I will rely on others for proper evaluation of this section.

Results

Line 291: Here and throughout the text, I think it would be more appropriate to refer to the intensity as “supra peak work rate” or something like that, so that it is clear that maximal work rate has not been determined in any of those studies. The same idea would apply to the term “submaximal”.

Line 298: I am happy to see that the authors used the right term here and referred to “peak work rate”.

Line 301: By design, this so-called verification phase cannot confirm the attainment of VO2max. As discussed earlier, the VO2max response remains a mystery. From my perspective, I would argue that VO2max was most likely achieved. However, given that the model used in many studies does not even allow for the VO2 response to be fully expressed, then this method risks falsely accepting the attainment of VO2max simply because the “supramaximal” intensity was too high to allow for the duration of exercise to be long enough for the VO2 response to be fully expressed (sorry for the extra-long sentence!). I would strongly recommend that the authors stay away from the idea of confirming that VO2max was attained as this is an untestable hypothesis. However, the authors can try to verify whether the VO2 associated with the “verification” ride was lower/higher than, or similar to, the VO2 observed during the incremental test.

Line 326: I like the wording here, as the author refer to the highest VO2. There might be value in knowing whether the verification phase results in a greater VO2 compared to the incremental test. However, as I indicated earlier, it is important not to confuse that with determination of VO2max.

General comment: I think that a further analysis that could be added would include the duration of the verification phases. I guess it will not change much as the mean data will hide the potential effect of duration. However, it is likely worth exploring whether shorter verification phases resulted in comparatively lower VO2 values.

Discussion

Line 417: As I indicated already, the verification phase cannot confirm the achievement of VO2max as the use of this approach is physiologically flawed. Thus, this meta-analysis should re-focus its aim and indicate a testable objective (i.e., identifying whether the VO2 during the verification phase is different from that obtained during the incremental test).

Lines 428-432: This section seems almost out of place or, at least, unnecessarily long. I understand that the authors might want to highlight the safety of the procedure in other populations. However, I would argue that the point of safety can be made in relation to the current data, and then the authors could add that several studies in clinical populations also confirm the safety of the procedure.

Line 434-435: Well, I guess my position on this wording is already clear to the authors. However, I will repeat it. This statement is unwarranted as no confirmation of VO2max has been obtained. Such confirmation requires a different experimental model as the intensity used in most verification phases are: 1) often too high for allowing a full development of the VO2 response; 2) below or at the peak PO, which invalidates the premise of "supramaximal" intensity to achieve the plateau criterion. In the end, the model helps you feeling more comfortable with the idea that the performance was maximal. However, the same limitations that applied to not feeling comfortable with a maximal effort during the incremental test also apply during the verification phase. Considering this and the idea that a plateau response is just an illusion (i.e., constant work rate loads become "supramaximal" much earlier than the peak PO observed during incremental testing), then the wording needs to be changed. Thus, the authors could say that "the verification phase is a safe and well-tolerated procedure to evaluate if a higher VO2 compared to that observed during the incremental test was obtained", or something in line with that.

Lines 441-443: These lines include some information that is correct, and some that sound wrong (at least as presented). It is true that incremental tests do not allow for the VO2 slow component to be fully expressed (with this being more noticeable with steeper ramps), which dissociates the relationship between VO2 and PO when comparing incremental and constant work rate exercise. However, the authors mentioned that the VO2 response is accelerated towards the end of the incremental test. This is incorrect. In fact, during the most used ramps (i.e., 20-30 W/min), the increase in VO2 for a given increase in PO is reduced towards the end of the ramp. This is reflective of the inability of the VO2 response to adjust fast enough to the rapidly increasing ramp. In fact, what the authors mentioned is observed during slow ramps (i.e., 5-10 W/min). This can be seen in Iannetta el al. (J Appl Physiol. 2019 Dec 1;127(6):1519-1527). Interestingly, that paper showed consistent VO2max responses with a wide range of peak POs, and a follow up paper showed that POs do not need to be "supramaximal" to confirm that the highest VO2 has been obtained (Am J Physiol Regul Integr Comp Physiol. 2020 Jul 22. doi: 10.1152/ajpregu.00126.2020). In fact, "supramaximal" intensities are discouraged. Importantly, the current data confirm that the incremental test is sufficient to obtain the highest possible VO2, which demonstrates that a verification phase does not offer much additional value.

Lines 444-449: This is perfectly in line with what I had just indicated. Given the wide range of ages and fitness levels, and the variety of ramps (i.e., 15, 20, and 25 W/min), the phenomenon that I just described is to be expected. In other words, slow ramps are far more likely to result in a plateau in the VO2 response as the slow component can be expressed to a larger extent, even within a narrower range of POs, as compared to fast ramps.

Lines 449-450: Exactly! If you think about it, the same ramp would be a relatively faster ramp for a less fit person compared to a fitter one (i.e., less time for the VO2 response to fully adjust).

Lines 465-476: This section could be just deleted as, aside from the use of HR responses, no other secondary criteria were considered in this analysis. Given that the discussion is excessively long, I would remove this section.

Lines 491-497: In fact, this information is in line with a recent paper from DiMenna's group (Arad et al., PLoS One. 2020 Jul 6;15(7):e0235567.doi: 10.1371/journal.pone.0235567). In this study (which should also be added to the analysis in my view), they showed that the verification phase resulted in a significantly (albeit minimally) greater VO2 compared to the incremental response. Interestingly, the authors commented that they selected 100% of peak PO to make sure that the duration of the verification phase was not too short. Based on the definition by Poole and Jones, the authors say that they cannot claim that VO2max was achieved because the intensity was not "supramaximal". However, they know that this idea of "supramaximal" is flawed anyway. Regardless, it is likely that a verification phase can help achieving a higher VO2 compared to the ramp test in some very specific circumstances. However, there is still no proof that VO2max has been achieved!

Lines 499-516: Well, this section is difficult to interpret as the results from Colakoglu's work are suspicious. How can you reconcile the results presented in Figure 2 in that study? I would be the first to admit (and I have made this point already) that 110% of the peak PO might result in a short duration for the verification ride, which might not allow for the VO2 response to be fully expressed. However, a near 20% drop in the VO2 response during the 110% verification ride is unthinkable! In fact, I went to the original study as the difference between the incremental test VO2 and the 100% verification ride VO2 was extremely high. After seeing the even greater difference at 110% of peak PO, I simply cannot trust those data (which is likely the reason why the paper is published in a very low impact journal). Regardless, the point is that the data from this study should probably not be considered. However, we are not the judges of science and I think it is fair to include the published results. That being said, a discussion on the surprising ~30% difference between the highest and lowest VO2 should be presented.

Lines 517-519: This should be reworded. What effectiveness has been demonstrated? In the end, the verification phase did not produce any significant differences in the results. Thus, rather than effective, I would consider this a waste of time. Based on your data, the recommendation should be that a verification phase is not included as it does not add any value to what was already informed by the incremental test. At best, you can say that "the verification phase procedure did not result in a higher VO2 response than that observed in continuous ramp or pseudo-ramp CPET protocols". Perhaps, you might want to feel that this adds validity to the procedure, and I can accept that as a personal preference. However, by no means can the authors say that VO2max was confirmed.

Lines 520-521: Why do the authors in theory agree with this premise? The premise of a "supramaximal" verification phase as a means of establishing the plateau criterion is physiological flawed. I hope that the authors can appreciate that now. In fact, this pre-conception sounds as a bias that pushes the authors to try to accept a perceived reality, even though the data

that they presented showed the opposite. I honestly think that the authors are to be commended for the detailed analysis that they did, and I truly think that the data are useful. I just cannot understand the origin of the authors' convictions on this topic.

Line 529: Here, the authors refer to "the utility of the verification phase". I believe this wording, although vague, is the closest you can get to reality. Anything that links the verification phase to a confirmation of VO2max is not scientifically verifiable using this model. Thus, this goes in line with what I have been saying from the beginning of my comments.

Lines 532-534: This is simply incorrect. I mean, that your results coincide with those of Poole and Jones is not. What is incorrect is that the verification phase provides evidence that VO2max was achieved.

Lines 535-540: As the authors might suspect, I do not agree with this overall statement. Any reference to this approach as a confirmation of VO2max should be avoided. It can be said that this was the goal of the approach, but that this goal cannot be achieved with this model. Referring to it as the "gold-standard" approach is simply ridiculous.

Lines 545-547: I would argue that the authors in that study should not limit their interpretation to older, less experienced or unfit participants. I mean, if the need for a verification phase is often purported to be connected to the idea that some participants might not be willing to complete a maximal effort during the incremental test, the same argument could be used as a reason to suspect that a maximal effort would not be performed during the verification phase. In other words, why would someone who is unwilling to push hard during the incremental test would suddenly become willing to push hard during the verification phase. In my view, this is another reason why thinking that the verification phase confirms VO2max is erroneous. First, the idea of a "supramaximal" intensity is flawed. Second, a verification phase cannot ensure that a supposedly absent maximal effort during the incremental test is now performed during the verification phase. In fact, if a suboptimal performance occurs during the verification phase, many would erroneously interpret that as proof that VO2max was achieved!

Line 551: This is correct and, as indicated above, the results from that study are highly irregular. I would attribute the differences to poor measurement rather than to physiological variability. A ~30% difference between the two extreme conditions is simply unacceptable.

Lines 551-557: Perhaps, neither "a)" nor "b)" are the correct interpretation. What your data clearly show (and I commend the author for doing an impressive work with this), is that the highest VO2 values during the incremental test is not different from the highest VO2 value during the verification phase. In neither case achievement of VO2max can be confirmed. If you ask for my personal opinion, I would argue that VO2max was achieved, and that the verification phase helped demonstrating that a supra critical intensity bout to exhaustion during the verification phase resulted in the same highest VO2 value as seen during the incremental test. In that sense, I am confident that the verification phase, although unnecessary in this population,

adds confidence to the measure. However, given the reasons explained before, the plateau criterion based on a “supramaximal” intensity is flawed, specially when the intensity for the verification phase is too high for the VO2 response to be fully expressed (which would lead to the wrong interpretation that VO2max has been confirmed).

Lines 558-560: I would argue that this research has been done (Am J Physiol Regul Integr Comp Physiol. 2020 Jul 22. doi: 10.1152/ajpregu.00126.2020) and that it should be incorporated into this manuscript.

Lines 563-564: As I mentioned earlier, the terms “supramaximal” and “submaximal” should be changed. In fact, defining the terminology would be important in relation to some of the concepts that I discussed before.

Lines 561-569: I like this paragraph because it highlights that any effort to exhaustion that is high enough above the critical intensity of exercise will result in the achievement of VO2max. In fact, if someone’s goal was just to measure VO2max, any “aggressive” constant work rate to exhaustion should be good enough (however, the goal of the test is often more ambitious than just evaluating VO2max). What I do not like though is the final sentence. I do not think that the authors can recommend that “procedures that are within the scope of the reviewed studies” should be used. As I mentioned, it sounds to me as if anything above the critical intensity would do it. The problem is that people do not normally do a verification phase at 75% of peak PO from a 30 W/min ramp! Thus, the recommendation is not easy to justify.

Lines 570-576: I guess I do not need to say again why this is not correct.

Lines 576-578: This is great. Then, almost everything written in this manuscript and most of my justifications were unnecessary! If the authors accept this fact, and thus accept that “supramaximal” efforts are not necessary, then they should have presented this idea from the beginning (even in the introduction), as it simply demonstrates that the concept of the “supramaximal” effort satisfying the plateau criterion is nonsense!

Lines 585-589: Brilliant! I agree with this 100%. It is disappointing though that the authors are saving the valuable physiological information for last! Seriously, I think that, in the last two paragraphs, the authors have debunked the “supramaximal” work rate theory. I think that this meta-analysis should not only present data, but also discuss the topic in a more physiologically relevant manner. This important section comes just towards the end of an excessively long discussion.

Lines 622-632: I think this is all good for discussion. However, in line with previous comments, these different criteria would be useful, in this model, to determine whether the highest VO2 from the incremental test is different from the verification phase beyond normal variability and/or measurement error. Once again, the verification phase approach cannot determine that VO2max has been achieved as its premise is flawed.

Line 633: Please change wording. The verification phase does not verify VO2max.

Lines 633-642: In general, this paragraph is weak. The authors presented the HR responses, but the discussion is very limited. For example, the idea that the kinetics of HR is slower than the kinetics of VO2 is not as evident as the authors make it sound (there is more information on this than the single reference that the authors presented). This could explain the differences in some studies, but likely not in the majority of them. I would argue that a priming effect with already improved blood flow availability before the onset of the verification phase might also play a role (although this is just speculation). Additionally, given that having a lower HR during the verification phase did not affect the VO2 responses, then the sex, intensity, etc. effects become irrelevant in my view. To be honest, I feel that given that this study is about the verification phase for determining whether or not a higher VO2 value can be achieved during this process, I would simply delete the HR analysis as it does not add anything to the story. In fact, it makes it longer and less focused.

Lines 655-672: The conclusion in general is too long as reads more as a summary than a conclusion.

Line 660: This is incorrect. Your analysis did show that different procedures can be applied to establish the same VO2 response during the verification phase as compared to the incremental test. However, by no means you can say that this procedure contributed to establish a true VO2max response (not even if using quotation marks for the word "true").

Lines 660-662: As I indicated before, what this meta-analysis highlighted is that any hard-enough intensity of exercise (i.e., "respectably" above the critical intensity) performed for long-enough will result in the highest possible VO2 response. Then, the recommendation that the verification phase should be constrained to the ones seen in the current analysis is unfair to me (or at least unnecessary). By doing this, the authors provide some level of validity to the proposed procedures and, indirectly, take validity away from other options that might be equally effective.

Lines 662-666: This makes no sense to me as reaching the same HR response is not a prerequisite to reach the highest possible VO2.

Lines 668: No, this verification phase has not validity as proof that VO2max has been achieved. It simply demonstrates that a higher VO2 value cannot be achieved during the verification phase. I insist in the idea that I would argue that this was, in fact, VO2max. However, that was already known from the incremental test (which is clearly shown in your data). What I oppose is the wrong idea that VO2max can be confirmed because the plateau criterion has been met. This is clearly not the case as the model is inappropriate to show that. Some people in the scientific community seems to lack the understanding of the differences between constant work rate and

incremental exercise responses, which has caused this misinterpretation of the verification phase as a tool to satisfy the plateau criterion.

Lines 668-670: This is correct. The verification phase did not add anything but some level of confidence that people pushed hard enough. I am not against that thought (as long as people do not confuse this with the idea that confirmation of VO2max has been achieved).

Lines 670-672: This is not a conclusion from the present study, but rather an opinion. I would delete this.
