# Supplementary material for: Is a verification phase useful for confirming maximal oxygen uptake in apparently healthy adults? A systematic review and meta-analysis
Source: PLoS One. 2021 Feb 17;16(2):e0247057. doi: 10.1371/journal.pone.0247057 (PMC7888616; doi:10.1371/journal.pone.0247057)
Supplement: Attachment — Submitted filename: PlosOnes_response letter.docx [file pone.0247057.s005.docx]

- Title: Verification phase for confirming maximal oxygen uptake in apparently healthy adults: A systematic review and meta-analysis

- Corresponding Author: Felipe A. Cunha

- E-mail: felipeac@globo.com

- Manuscript ID: PONE-D-20-25408R1

Dear Editor,

Please find below our responses to the reviewers’ comments concerning the article PONE-D-20-25408, titled “*Verification phase for confirming maximal oxygen uptake in apparently healthy adults: A systematic review and meta-analysis*”. The manuscript has been revised according to the reviewers’ suggestions and an itemized, point-by-point response to each of the reviewers’ comments has been provided.

Yours Sincerely,

Felipe A. Cunha

Review comments:

**Reviewer: 1**

I would like to thank the authors for their attention to my comments. I think this revised version is much better and I believe that this manuscript would be an excellent reference on the topic. From my perspective, I only have on minor suggestion that I would leave up to the authors to accept or reject. This is: As presented, the title seems to imply that a verification phase is something necessary. Would it be better crafting the title as a question? Something like “Is a verification phase for confirming maximal oxygen uptake useful in apparently healthy adults: A systematic review and meta-analysis”.

Regardless, I would like to congratulate the authors for the high quality of this meta-analysis. I often feel that this type of scientific contributions are not as meritorious as original research, but I think that with the amount of information that is currently available on this topic, this particular meta-analysis is fully warranted. Thanks!

*ANSWER*: Thank you for your comments. The title has been revised according to the reviewer’s suggestion.

**Reviewer #2:**

General Comments

Preparing this manuscript has clearly been a huge job, so I commend the authors on this significant undertaking. I have again reviewed this paper in thorough detail and have a number of observations and feedback points, specified below.

In general, I would firstly urge the native English speakers on the author list – or a professional proof reader – to take responsibility for thoroughly checking the language (especially the grammar) before resubmitting, as the current level of writing makes the text difficult to comprehend. There are particular issues in the new sections of the Discussion that need re-writing/correcting to improve the clarity and flow.

Secondly, and perhaps most importantly, the Discussion to me is too long. It makes it impossible for the reader to grasp the key findings and messages from this paper, as there is just so much detail of previous studies, their protocols and specific findings (data, P values, etc.). I personally feel that the paper would be far more comprehensible and impactful if the Discussion was more concise.

*ANSWER*: The discussion section has undergone a comprehensive revision to improve the grammar and make it more concise by removing specific details that provide limited information for the interpretation of the results.

**Abstract:**

L33-35: The use of semicolons in this list seems odd, and makes it difficult to understand how the search was conducted. Should they be commas? Please re-consider.

*ANSWER*: The correction has been made.

L43: Why the comma after VO2max but not after age? Also, the -1 on the VO2max unit looks too high.

*ANSWER*: The correction has been made.

L44: Can you clarify that the VO2 values were similar in 54 of the 80 studies, because at the moment it seems like this is a result for a total of 54 studies analyzed, which I don’t think is the case. Suggestion: “The highest mean VO2 in the CPET and verification phase was similar in 54 of the 80 studies (mean difference…)”. Also, I still don’t understand the inconsistencies in the use of square/round brackets. Can this be standardized throughout the manuscript? Also, why change to L/min in this sentence, after presenting mL/kg/min in the previous sentence? Can this be standardized?

*ANSWER*: Thank you for your comment. We have revised the paragraph according to the reviewer’s suggestion and it now reads as follows: *“**The highest mean VO_2_ values attained in the CPET and verification phase were similar in the 54 studies that were meta-analyzed (mean difference = 0.03 [95% CI = -0.01 to 0.06] L/**min, P = 0.15)*.*”* In addition, we have standardized the use of square/round brackets throughout the manuscript. On the other hand, VO_2max_ relative to body weight has been used as descriptive data for defining levels of cardiorespiratory fitness for sub-analysis, whereas absolute values of VO_2_ have been used for the meta-analysis since most studies assumed L/min. We have now also included mean ± SD VO_2_ values in L/min in the abstract as follows: “*Eighty studies were included in the systematic review (total sample of 1,680 participants; 473 women; age 19-68 yr.; VO_2max_ 3.3 ± 1.4 L/min or 46.9±12.1 mL·kg^-1^min^-1^)*.”

L51: “following a… CPETs” = incorrect grammar. Maybe write CPET

*ANSWER*: The correction has been made.

L51-53: I like this idea, but to me it needs to be related specifically to “your” findings, not attributed to “some [other?] researchers”. Maybe something like: “However, given the high concordance between the highest mean VO2 achieved in the CPET and verification phase, findings from the current study would question its necessity in all testing circumstances.”

*ANSWER*: This sentence has been rewritten according to the reviewer’s suggestion.

**Introduction:**

L70: Maybe mention the Douglas bag method here, since that is the main reason for the improved time-efficiency (i.e., changing from DBs to B-b-B systems). The type of protocol is merely a by-product.

*ANSWER*: The correction has been made.

L73-74: This sentence is not quite right. The “confirmation of VO2max attainment” is not “due to” the listed factors; the “lack of attaining” VO2max might be due to those factors. Please re-phrase.

*ANSWER*: Thank you for the observation. The correction has been made. The sentence now reads as follows: “*One particularly problematic aspect has been the challenge in identifying a lack of VO_2max_ attainment due to inappropriate test protocols, premature fatigue, or poor participant motivation and lack of effort*.”

L82: Presumably this is average “male” body mass. Please specify (there needs to be transparency when sex biases exist in data sets).

*ANSWER*: The reviewer is correct, thank you for the comment. The following information has been included*: “The landmark study of Taylor et al. [34] was the first to use a formal VO_2_ plateau criterion, which was defined as an increase in VO_2_ of less than 150 L/min (or ≤ 2.1 mL·kg^-1^·min^-1^, considering an average body mass of 72 kg from 115 male subjects) (…)”*

L85-89: Is this a new paragraph? It seems very short (2 sentences). Maybe it should be a continuation of the previous paragraph, since it’s the same topic (VO2 plateau)?

*ANSWER*: It is the same paragraph that was summarized to attend both reviewers. Lines 78 to 89 describe the same topic – VO_2_ plateau. This is more clearly shown in the revised manuscript.

L93: “is attained”?

*ANSWER*: We have revised the text to “*has been attained*” since VO_2max_ criteria are applied after the VO_2max_ test has been performed.

L95-96: I’m not sure what this means: “lack of specificity in identifying individuals who did not continue the CPET to their limit of exercise tolerance”. What is meant by “specificity” in this context?

*ANSWER*: The following sentence has been included to address this issue: “*Research has shown that some individuals can satisfy some of the secondary criteria thresholds long before the highest VO_2_ value observed in the CPET has been attained [2, 29, 37, 39]. The maximal RER criterion, for example, can be satisfied at VO_2_ values 27%-39% lower than the highest VO_2_ value achieved in the CPET [37, 39]*.”

L103: CP is specific to power (usually analogous to cycling) and not practically applicable to other exercise modes (such as running). Also, “i.e.” is specific (“that is”). So maybe change i.e. to e.g. (“for example”). Or add speed/velocity to power when referring to CP (so CS/CV).

*ANSWER*: The correction has been made.

L106: Suggestion (to avoid the use of “each other”): “the highest VO2 values in the CPET are consistent with the verification phase”

*ANSWER*: The correction has been made and now the sentence reads as follows: “*The verification phase is based on the premise that when the highest VO_2_ values in the CPET are consistent with the verification phase (typically within 2-3% in accordance with the test-retest reliability of VO_2max_), this provides substantial empirical support that the highest possible VO_2_ has been elicited*.

L108-111: Please present the “evidence” for Poole & Jones’ statement (i.e., “that a verification phase must be performed at a higher WR than attained in the ramp-based CPET protocols in all future studies”). Where/what is the evidence for this suggestion?

*ANSWER*: We described the exact term employed by Poole and Jones (2017). However, to avoid further controversy on this issue, we decided to replace “*must be*” to “*should be*”.

L111: The terminology “On the other hand” seems inappropriate, as this is a different topic compared with the previous sentence (VP WR vs CPET increments). The correct counter-argument here would presumably be the data showing that VP WR can actually be “lower” than WRpeak to still elicit a VO2peak/max.

*ANSWER*: This section of the introduction has been considerably revised and includes addressing this issue.

L113-116: This is now confusing, with “In contrast” following “On the other hand”. Please clarify the arguments that you are making, and the key information you’re trying to get across. I also think WRpeak needs defining. Also, is it necessary to list average W values?

*ANSWER*: The text has been revised as follows: “*Poole and Jones [2] recently stated that to confirm the attainment of VO_2max_ a verification phase should be performed at a higher WR than attained in the CPET in all future studies. Conversely, Iannetta et al. [25] recommended WRs within the upper limit of the severe exercise intensity domain to allow the verification phase to be maintained long enough for VO_2max_ attainment.*”

L108-120: In general, this section needs to be more clear and concise.

*ANSWER*: This section has been revised for conciseness, so it is now only 8 lines.

L120-132: I think all of these ideas can be incorporated into one far more clear and concise paragraph explicitly stating the aims of the study. I suggest moving the questions (at L120-125) down to L138-141 (Methods) and just focusing on the aims here, as required.

*ANSWER*: This text has been revised according to the reviewer’s suggestion.

**Methods:**

L136: “is” shown in S1 Text.

*ANSWER*: The correction has been made.

L138-141: These are not completely consistent with the text in the Introduction (L120-132). Also, you are still using the terminology “valid alternative to confirm”, which I understood had been changed throughout (since this is not a study of validity). And I’m not sure the study does actually identify “the most appropriate protocol for applying the verification phase”. So I would use questions similar to those currently posed in the Introduction (L120-125) here instead.

*ANSWER*: The correction has been made according to the reviewer’s suggestion as follows: “*The main questions addressed by the present study were: To what extent does the highest VO_2_ attained in the CPET differ from that attained in the verification phase? Secondly, are the highest VO_2_ values in the CPET and verification phase affected by the verification phase characteristics (e.g. intensity, adoption of a criterion threshold, and aspects of the recovery period between the CPET and the verification phase), or even with respect to particular subgroups (e.g. sex, cardiorespiratory fitness levels, exercise test modality, and CPET protocol design) in apparently healthy adults?*”

L155: participants “who” were

*ANSWER*: The correction has been made.

L157-158: Suggestion: “…carried out using bipedal cycle ergometry or bipedal treadmill running or walking.”

*ANSWER*: We agree with the reviewer’s comment and the sentence has been rewritten accordingly.

L160: Remove “included” (as you already have “involved”, above). Also, importantly, “and” should presumably be changed to “or”, since any one of those three situations would surely lead to exclusion.

*ANSWER*: The correction has been made.

L166-167: Writing in the first person seems odd here (we). Are you saying that a flowchart has been included in the paper? If so, where is it? If not, why mention it?

*ANSWER*: The correction has been made.

L170-174: There is still an inconsistent use of round and square brackets. Please re-consider (round brackets are typically used in the first instance, and [square brackets] within round brackets.)

*ANSWER*: We have standardized this throughout the manuscript according to your suggestion.

L175: Can you specify that you mean “other” authors than yourselves, so “authors of the original articles were contacted…”.

*ANSWER*: Thank you for the observation. We have revised the paragraph according to the reviewer’s suggestion and now it reads as follows: “*When the relevant quantitative data were not reported, authors of the original studies were contacted to request the data*”.

L185: Does “selective reporting” need to be written twice here?

*ANSWER*: We have shortened it to “*selective reporting of outcomes*”.

L187: You are inconsistent with your capitalization (or not) of the sub-title words

*ANSWER*: The capitalization of the sub-title words is now standardized.

L206: “the primary study groups results” requires an apostrophe somewhere on groups, depending on the specific meaning (one group or multiple groups) – please correct.

*ANSWER*: The text has been revised to address this issue and now reads as follows: “(*mean ± standard deviation [SD] values for group VO_2max_ and protocol duration during the CPET and verification phase from primary study groups)*”.

L207: Same here – “groups” needs an apostrophe.

*ANSWER*: The text has been revised to address this issue and now reads as follows: “*The I^2^ statistic measures the extent of inconsistency among the results of the primary study groups, interpreted approximately as the proportion of total variation in point estimates that is due to heterogeneity rather than sampling error.*”

L208: The less than or equal to symbol is still odd here. The lower “equal to” line should be horizontal.

*ANSWER*: The correction has been made.

L209: using “a” funnel plot, or using funnel “plots”?

*ANSWER*: We have added the word “a” funnel plot. Thank you for the observation.

L216: This new red text is unnecessarily complicated. Firstly, you have previously used the term WRpeak (although this doesn’t seem to have been defined anywhere in the manuscript). Secondly, sub and supra peak does not need to be reinforced with < or > 100%, that’s obvious by definition. Suggestion: (i.e. < 100% WRpeak vs. > 100% WRpeak)

*ANSWER*: The correction has been made.

L218-220: This sentence could be clearer, suggestion: “as the CPET or on a different day, and the duration of the verification phase (i.e. ≤ 80 s, 81–120 s, > 120 s).”

*ANSWER*: The correction has been made.

L222: Should this be “cut-off points”?

*ANSWER*: The correction has been made.

**Results:**

L229-230: This (“Figure 1 summarizes the screening and selection process”) seems like repetition from the Methods section (L166-167). Should the flowchart and reference to it be moved up to the Methods (L167)?

*ANSWER*: The flowchart has been moved up to the Methods section.

L234: Presumably your sub-section headings should use a different font/presentation from your main section headings.

*ANSWER*: The sub-titles have been standardized throughout the manuscript.

L235-239: In L235 you use the term “eligible studies”, in L236 you write “included studies” and in L239 you write “primary studies”. This is confusing, because I think you are talking about the same 80 studies in all cases. Can you use consistent terminology for clarity?

*ANSWER*: “Primary” has been removed and “eligible” has been replaced by “included” to keep the text standardized. You are correct, this means the same 80 studies.

L240-241: BMI has already been defined in the paper, so I suggest: “participants had a BMI within the normal range (mean ± SD [range]: 24.4…”. And once again, you need to be consistent with the use of round/square brackets.

*ANSWER*: We have incorporated your suggestion and square and round brackets are now standardized throughout the manuscript.

L242: VO2max does not need to be written twice; I suggest removing the first reference to it: “cardiorespiratory fitness (VO2max mean ± SD [range]: 46.9…”.

*ANSWER*: We have incorporated your suggestion.

L243: Delete “according to… [53]” as these have already been defined in the Methods (L222-223).

*ANSWER*: This sentence has been removed.

L252-256: I think this could be clearer, e.g.: “Thirty-three (41%) of the 80 studies included in the review adopted one or more of the traditionally reported plateau or secondary criteria to confirm the attainment of a VO2max, with 30 using a VO2 plateau criteria, 21 using the heart rate plateau or age-predicted maximal heart rate, 18 using RERmax, and 8 using post-CPET blood lactate concentration.”. Line 256 is missing a full stop in any case.

*ANSWER*: We have attempted to make this text clearer. The text now reads as follows: “*Thirty-three (41%) of the 80 studies included in the review used one or more VO_2_ plateau or secondary VO_2max_ criteria to confirm the attainment of VO_2max_. Thirty studies used the VO_2_ plateau**, 21 used the heart rate plateau or a criterion based on age-predicted maximal heart rate, 18 used the RER_max_, and 8 used the post-CPET blood lactate concentration.*”

L258: I don’t think the second “time averages” should be hyphenated.

*ANSWER*: The hyphen has been removed.

L263: Should this read something like “Regarding the period between CPET and VP…”? Because you seem to be referring to the rest period after the CPET here, right?

*ANSWER*: We have incorporated your suggestion.

L266: What do you mean by “self-paced approach”? A self-paced recovery period? Of any duration?

*ANSWER*: This text has been revised and now reads as follows: “*Two studies (3%) employed a combination of passive and active recovery and another (1%) used a self-paced approach where participants were permitted to choose their own WR.*”

L270-271: You need to be clearer that you are now referring to the VP exercise intensity. I also think you should decide upon the terminology earlier in the paper, define it and stick to it (e.g. peak WR or WRpeak, supra or > 100%, etc.). Because at the moment there is a mix.

*ANSWER*: The revised text now specifically refers to the verification phase. Consistent terminology also has now been used for supra in instead of > 100% and for WR_peak_ instead of peak WR.

L272: Maybe write 105-130%, consistent with the presentation of ranges in the previous paragraph.

*ANSWER*: The correction has been made.

L273: What do you mean by “or both peak or supra peak WR (1%)”? Is this one study, a different study from the “Eight studies” previously stated? If so, please specify. Also, do you mean both peak “and” supra peak? This is unclear to me.

*ANSWER*: The study of Wingo et al. (Medicine & Science in Sports & Exercise, v. 37, n. 2, p. 248-255, 2005) stated the following sentence “*To ensure that a plateau in VO_2_ was attained, subjects completed an additional bout of cycling following 20 min of rest. Subjects cycled to exhaustion at a power output equivalent to the last workload performed during the graded test (if ≤ 1 min was completed during the last stage of the graded test) or at a power output 25 W higher than the last workload performed during the graded test (if ≥ 1 min was completed during the last stage of the graded test)*”. This means that the usage of either peak (100% WR_peak_) or supra peak WR (>100% WR_peak_) depended on the duration of the last CPET stage. Another study (COLAKOGLU, Muzaffer et al. Stroke volume responses may be related to the gap between peak and maximal O_2_ consumption. Isokinetics and Exercise Science, v. 24, n. 2, p. 133-139, 2016.) also applied both peak and supra peak WRs. Considering this, the sentence has been rewritten and we think it is now clearer.

L275: Again, does 1% relate to one study? If so, you could maybe write (one study, 1%) for clarity/consistency. Also, you have previously used the term “participant” rather than “subject”.

*ANSWER*: “*One study*” has been added. “*Participants*” is now used consistently throughout the manuscript,

L277: “85-95% of…”

*ANSWER*: The correction has been made.

L281-282: “1.5-2.2”; ”50-150” (consistent with previously stated ranges).

*ANSWER*: The correction has been made.

L294: Figure 2 (not Figures).

*ANSWER*: The correction has been made.

L322: I suggest removing the two commas, as this “middle” information is imperative to understanding the analyses.

*ANSWER*: The correction has been made.

L338-339: Please add units to the IQR, presumably s.

*ANSWER*: The units have been added.

L339-340: This very last line of the Results section: “There were no significant differences between the CPET and verification phase for VO2max (P = 0.18 to P = 0.71)”… Can you be more specific about what the sentence, and the range of P values, relate to. For example, how does the information differ from that stated in L295-297 (i.e., “Notably, the mean highest VO2 values were similar between the CPET and verification phase [mean difference = 0.03 (95% CI = -0.01 to 0.06) L/min, P = 0.15].”)? Presumably you are referring to the sub-groups, but it’s currently unclear from what you have written.

*ANSWER*: We have added the following sentence “*Considering all sub-analyses presented in the Table 4…*”. By looking at the Table 4, we can see the minimal *P* value (0.18 for the high cardiorespiratory fitness) and the maximal *P* value (0.71 for female subjects).

**Discussion:**

In general the writing in this section, particularly the new parts in red, need reviewing and revising to improve the clarity of the messages being communicated. The grammar is poor in places, which makes the important information difficult to understand. Also, the Discussion to me is far too long. There are unnecessarily lengthy descriptions of studies and protocols throughout, so I suggest writing more concisely. Also, there are lengthy descriptions relating to points not supported by the current data analysis. In my mind, the authors need to make a significant overhaul of the discussion and cut it down in length, such that the important points and messages are communicated far more clearly.

*ANSWER*: The discussion section has been comprehensively revised to improve the grammar and make the it more concise, including removal of the unnecessary descriptions of studies.

L357-359: The word “repeated” appears out of place here, since the VP is not repeated (it is only carried out once). Also “compared to that observed” is unclear – what are you comparing to? The maximal effort and VO2max (in which case, plural = those)? Please review and revise this sentence for clarity.

*ANSWER*: The correction has been made. We have replaced “repeated” by “carried out” and “that” by “highest VO_2_” observed in the preceding CPET, which is the dependent variable measured in the two protocols.

L360: “To date” should not be hyphenated (the term also seems odd/unnecessary). Also, consider re-phrasing: “has demonstrated that… have shown that…”.

*ANSWER*: Thank you for the observation. We have removed “*to-date*” from the sentence.

L364: “findings from 54 CPET…”. Also, I don’t really understand what this sentence adds to the previous sentence. They seem to say the same thing. Can they be combined?

*ANSWER*: The first sentence refers to results from the systematic review that includes all 80 studies, while the second sentence refers to the meta-analyzed data that includes only 54 of the 80 studies). It means that the qualitative results (systematic review) support the quantitative ones (meta-analysis). A hypothetical situation could happen whereby data from the systematic review could indicate VO_2max_ attainment in both protocols that are not be confirmed through the statistical analysis. Although the two sentences appear similar, they are reinforcing the consistency of results from a different amount of studies in each case (80 *vs.* 54).

L366: Please revisit the grammar and revise this sentence.

*ANSWER*: This sentence has been removed from the revised discussion section.

L369-370: I suggest removing this important information from the brackets and supporting the statement with a reference (i.e., that this is within the most commonly adopted measures of test variability at 2-3%).

*ANSWER*: The correction has been made.

L371: Revise the grammar.

*ANSWER*: This text has been removed from the revised discussion section.

L374-375: Revise the grammar.

*ANSWER*: This text has been removed from the revised discussion section.

L383: “for example” appears out of place here, in a new paragraph.

*ANSWER*: This text has been removed from the revised discussion section.

L383-393: This is a long account of a previous study. What point is being made?

*ANSWER*: This relevant study by Day et al. reported that traditional VO_2max_ criteria are protocol dependent (the plateau occurrence is not a commonly observed phenomenon in continuous step-incremented tests and even less so in individuals with low cardiorespiratory fitness). In contrast, a verification bout induced no difference in VO_2_ compared to that observed in the CPET. In summary, the verification phase does not appear to be affected by protocol design and is not population dependent. This is an important point and the findings by Day et al. are consistent with the results from the current meta-analysis, because all sub-analyses indicated no differences according to protocol and individual characteristics.

L383-403: This is a long paragraph describing a few previous studies and their results, but there is no reference to the overall findings of current study. Please revise.

*ANSWER*: Rossiter et al. investigated similar questions as those by Day et al., which already have been commented on in our previous answer.

L404: A new paragraph should not begin with “On the other hand”. Also, is this point (“54 studies meta-analyzed”) now in relation to your meta-analysis? Please clarify. In general, I suggest you revise your paragraph structures and arguments to arrive more quickly at the points you are trying to make, in light of previous work, and link the story to the findings of the current analysis. At the moment I am wondering where all this is going (particularly the paragraph above), and what the relevance is to the findings of your systematic review/meta-analysis.

*ANSWER*: “*On the other hand*” has been removed from the sentence. The 54 studies are from our meta-analysis. The next paragraphs discuss a small quantity of the included studies (~11%) that found significant mean differences between the CPET and verification phase. Moreover, we tried to explain the possible reasons for these differences. Forty-eight out of 54 studies showed, by analyzing individually, no difference in the highest VO_2_ between the CPET and verification phase, being consistent with the present meta-analysis. We have comprehensively revised the discussion section to more quickly arrive at the points being made to address the reviewer’s comment.

L406-409: The use of brackets seems inconsistent here too.

*ANSWER*: The round and square brackets are now standardized throughout the manuscript.

L425-426: Please revise the writing here: “since low cardiorespiratory fitness are more susceptible to stopping early during the CPET”

*ANSWER*: We have added “*individuals with*” low...

L404-432: This is another very long paragraph describing previous studies in length. I think these points can be made far more concisely and interpreted more clearly in light of the general findings from your study.

*ANSWER*: We have removed the unnecessary detail as part of the comprehensive revision of the discussion section.

L427-432: You seem to have spent a long time describing a possibility that is not actually supported by your own data analysis. I suggest turning your thinking around, and discussing what you did find and what that actually means, in relation to the specific studies analysed. I get your point, but it is not actually supported by your stats, which is the issue. Alternatively, make this point more concisely so that it is not over-inflated.

*ANSWER*: The percentages are from our data analysis. The difference across cardiorespiratory fitness levels was not significant, supporting the use of the verification phase regardless of individual characteristics, which contrasts with the low occurrence of the VO_2_ plateau during a CPET in subjects with low cardiorespiratory fitness.

L433: Due to the shear length of this discussion I have read the remainder with less of a focus on details. As stated above, my advice is that the writing is more clear and focused, with less extensive description of previous studies.

*ANSWER*: The discussion section has undergone a comprehensive revision to improve the grammar and make it more concise by removing specific details that provide limited information for the interpretation of the results.

L499-501: Again, is there any data to support Poole and Jones’ suggestion here (i.e., that researchers “must”…), or is it a subjective view (because other research would suggest that this is not necessary)?

*ANSWER*: The correction has been made.

Table 1:

Title: (N = 80) – include space; CPET has not been defined here, but it has in the Fig 2 title.

*ANSWER*: A space has been included and CPET has now been defined.

Table 2:

Abbreviations for TR and CYC seem to be missing (I haven’t read every detail of Table 2, so there might be other bits missing).

*ANSWER*: We have double-checked the abbreviations to ensure there are none missing.

Table 3:

Can you clarify in the table heading row what is meant by “Total”

*ANSWER*: “*Total*” means the number of participants that have performed both CPET and the verification phase. As we used Review Manager to calculate the statistics, the software layout requires input data in this order: mean, standard deviation and number of participants for each intervention.

For Arad et al., % Weight should probably be written to 2 d.p. (1.40%).

*ANSWER*: Thank you, the correction has been made.

Figure 1:

This is a nice figure. Is it what you refer to in L167?

*ANSWER*: Yes. This figure is a flowchart with the process of inclusion and exclusion of the studies.

“Hand searches (reference lists from the previously identified studies)”

*ANSWER:* Thank you. The correction has been made.

Should it be “Records excluded”? (Box to the right in “Screening”?)

*ANSWER:* Yes. We have changed to “Records excluded”.

Eligibility: can the horizontal arrow stem from the left box?

*ANSWER*: Figure 1 has been formatted according to PRISMA guidelines and we would prefer to keep it in its current format.

Figure 2:

Legend on L319: Suggest “reported as mean differences (MD) adjusted for” (and remove the final sentence on this line).

*ANSWER*: We have incorporated your suggestion.
