# Supplementary material for: Is a verification phase useful for confirming maximal oxygen uptake in apparently healthy adults? A systematic review and meta-analysis
Source: PLoS One. 2021 Feb 17;16(2):e0247057. doi: 10.1371/journal.pone.0247057 (PMC7888616; doi:10.1371/journal.pone.0247057)
Supplement: Attachment — Submitted filename: PlosOnes_response letter.docx [file pone.0247057.s006.docx]

- Title: Is a verification phase useful for confirming maximal oxygen uptake in apparently healthy adults? A systematic review and meta-analysis

- Corresponding Author: Felipe A. Cunha

- E-mail: felipeac@globo.com

- Manuscript ID: PONE-D-20-25408R2

Dear Editor,

Please find below our responses to the reviewers’ comments concerning the article PONE-D-20-25408, titled “*Is a verification phase useful for confirming maximal oxygen uptake in apparently healthy adults? A systematic review and meta-analysis*”. The manuscript has been revised according to the reviewers’ suggestions and an itemized, point-by-point response to each of the reviewers’ comments has been provided.

Yours Sincerely,

Felipe A. Cunha

**Reviewer #2:**

General Comments

Reviewer #2: Congratulations on producing a great paper! I have once again read the manuscript in detail and have a series of small suggestions. Most reflections relate to the Discussion, but I am happy to accept the manuscript following a final revision by the authors.

*ANSWER*: Thank you for your comments and for the obvious substantial time and effort you have given to help improve our paper.

Abstract:

Really clear.

*ANSWER*: Thank you for your comment.

Introduction:

Really informative. Just a few small suggestions…

L68: “fast-responding” – NOTE that this rule should be applied throughout the manuscript in my opinion… two words forming an adjective to describe a subsequent noun should be hyphenated (there are lots of examples, but e.g. verification-phase characteristics, verification-phase duration, highly-trained endurance athletes, test-protocol dependent, 10-min recovery, different-day variability etc.).

*ANSWER*: You are correct. The corrections have been made.

L100: 27-39%

*ANSWER*: The alteration has been made.

L117: Could you just double check whether WRpeak has already been defined. If not then I suggest you explicitly define the abbreviation here.

*ANSWER*: Thank you. We have included its definition: […] a verification phase should be performed at a higher WR than the last load attained in the CPET (i.e. > WRpeak) […].

Methods:

Really clear. Just a few small suggestions…

L135: Suggest writing the PROSPERO weblink and reg number in one single sentence.

*ANSWER*: We have incorporated your suggestion.

L161: This final criteria seems redundant when you have specified (at L158) “bipedal treadmill running or walking”. I would remove it (otherwise the reader could wonder why you haven’t included outdoor/track cycling as an exclusion criteria).

*ANSWER*: Thank you. We have removed the final criterion.

L204: You are missing a full stop.

*ANSWER*: A full stop has been added.

Results:

Really thorough. Some simple suggestions to consider…

L231-233: “All potential… for eligibility”. These two sentences seem like methods. I suggest removing them.

*ANSWER*: The two sentences have been removed.

L263-266: This makes 82 studies in total (103%). I suggest that the two studies using both are not included in the other lists, so that they are not duplicated (if that’s what’s happened) and so that the total = 80. If not then at least re-phrase “and another two”, because that’s not strictly true. It’s two of those already mentioned (so something like “two of which”). I would actually re-order the list to have continuous, then discontinuous, then both cont+discont, then self-paced, just to improve the logical flow for the reader. But it’s up to you!

*ANSWER*: We have revised the sentence according to your suggestion and it now reads as follows: “Seventy-three studies (91%) used continuous step-incremented or ramp/pseudo-ramp CPET protocols. Three (4%) used only discontinuous step-incremented protocols. Two studies (3%) used both discontinuous and continuous step-incremented protocols and another two studies (3%) applied self-paced protocols.”

L269: Could you double check that the RERmax abbreviation has been defined already. If not then I suggest you explicitly define it here.

*ANSWER*: We have included its definition: […] 18 used the maximal RER attained in the CPET (RERmax) […].

L271-275: It’s a bit unclear why you’ve included %ages for the first two examples, but not throughout the rest of the sentence (especially when %ages are included in the previous and following sentences). Maybe it’s all the small numbers, but something to re-consider.

*ANSWER*: We have added the percentages so every count in the paragraph has a corresponding percentage.

L293: Forty-two studies (53%) employed

*ANSWER*: The alteration has been made.

L296: researchers’ laboratories

*ANSWER*: The alteration has been made.

L313: were judged to have a low risk of bias…

*ANSWER*: The alteration has been made.

Discussion:

I have some minor suggestions and reflections…

*ANSWER*: OK.

L362-363: of these studies, 90% of which have been published since 2009.

*ANSWER*: The alteration has been made.

L363: I would probably remove “of the review”, because it’s both a review and a MA.

*ANSWER*: “Review” has been removed.

L367-369: I suggest removing these two sentences as both seem out of place, with neither relating directly to the aims of the study. Firstly, you didn’t explicitly investigate safety across fitness groups. And the second sentence implies that you could run a VP as a stand-alone test to measure VO2max, which you didn’t investigate (and shouldn’t be implied, as the effect of the previous CPET is a confounder).

*ANSWER*: These sentences have been removed.

L373: Is “error” the correct term here? Isn’t it just a “difference”?

*ANSWER*: We have changed “error” to “difference”.

L375: You didn’t measure agreement, specifically. Is a better term “similarity”.

*ANSWER*: We have changed “agreement” to “similarity”.

L379-382: Have you got this the wrong way round? Are low fitness groups not “less” likely to exhibit a VO2 plateau as a result of VO2 not decelerating?

*ANSWER*: You are correct. Thank you for spotting this error. The correction has been made.

L385: When you write “mean VO2 values”, do you actually mean “mean VO2max values”? There is of course an important difference between mean VO2 and mean VO2max. Also in this sentence, you should probably write “by” 0.03 and 0.04 L/min (not “of”).

*ANSWER*: We have written “mean VO_2max_ values”. The word “mean” means the highest VO_2_ the individuals could achieve. The correction has been made.

L386-389: This sentence is confusing. Suggestion: “However, sub-group analyses revealed that while maximal VO2 in the CPET was higher than that attained in the verification phase for participants with moderate and high cardiorespiratory fitness, the opposite was true for those with lower cardiorespiratory fitness.”

*ANSWER*: Thank you. We have incorporated your suggestion.

L394: Same here as in L385, do you mean “mean VO2max”, rather than just “mean VO2”?

*ANSWER*: We have revised the text to include “mean VO_2max_”.

L399: Regarding verification phase… (or “In regards to…”)

*ANSWER*: The text has been changed to “Regarding verification intensity…”

L402: who performed five repeated treadmill CPET trials appended by a verification phase…

*ANSWER*: The alteration has been made.

L406: I would remove the term “inappropriately”, unless you can support the inappropriateness clearly with evidence/a reference.

*ANSWER*: We have removed “inappropriately” as suggested.

L408-409: which allowed sufficient time (i.e., ~ XXX s) for VO2max attainment [can you include the VP duration here, to clarify how much greater it was than the previously highlighted 80 s].

*ANSWER*: The suggested information has been added.

L412-413: found no difference for verification phase durations of ≤ 80 s, 81-120 s, and > 120 s

*ANSWER*: The correction has been made.

L414: Again, where is the justification for deeming this duration “inappropriate”? Please provide evidence/a reference.

*ANSWER*: We have removed the term “inappropriate”, consistent with our response to your previous comment on this issue.

L422: 1-hr should not be hyphenated.

*ANSWER*: The correction has been made.

L423-424: What about fatigue?

*ANSWER*: We have added information on fatigue and the sentence now reads as follows: “It is feasible that the procedures performed before the maximal CPET may have led to poor participant motivation, lack of effort and premature fatigue in the following test”.

L426: The opposite is also plausible, though, as prior exercise (warm-up/priming) is known to have favorable effects on VO2. So I think you need to justify the greater advantage of no prior “fatiguing” exercise compared to no warm-up.

*ANSWER*: The aim of this sentence is to highlight the potential effect of residual fatigue from the maximal CPET on subsequent verification phase performance. We have added the word “maximal” to the sentence to help clarify this.

L436: Would “effectiveness” be better than “utility”?

*ANSWER*: We have incorporated your suggestion.

L440: Suggest inserting a comma after [128]

*ANSWER*: The comma has been inserted.

L441: Suggest removing the comma after “phase”

*ANSWER*: The comma has been removed.

L442: suggested that researchers…

*ANSWER*: The correction has been made.

L448: is sufficiently long.

*ANSWER*: The correction has been made.

L456-458: To me the problem seems to be more an issue of confounding results rather than limited data. There are loads of studies, as you’ve highlighted, but they all show different results!

*ANSWER*: We have changed “limited data” to “confounding results”.

L458: evidence-based

*ANSWER*: The alteration has been made.

L459: 10-20 min

*ANSWER*: The correction has been made.

L463: Where is the evidence that this “might be better tolerated”? Is there evidence that performing the VP on the same day is not well tolerated? I would remove this idea and just write “An alternative method is to perform the VP on a separate day…”

*ANSWER*: We have incorporated your suggestion.

L498: Suggest changing “patient” to “participant”, as this is not just relevant to clinical populations, but also healthy/athletes, etc.

*ANSWER*: We have incorporated your suggestion.

L500: I don’t understand the term “would be ideally indicated in”, in this context. Something like “is applicable to” might be better.

*ANSWER*: We have incorporated your suggestion.

L502: Does “those” refer to the wheelchair athletes? Presumably not, so maybe write more clearly: “individuals with spinal-cord injuries”.

*ANSWER*: We have incorporated your suggestion.

L504-505: This seems repetitive from L499-500, so I suggest removing this sentence.

*ANSWER*: We have removed this sentence.

Tables:

Table 3 heading: Would it be better as “Overall comparisons in the meta-analyzed studies…”

*ANSWER*: We have incorporated your suggestion.

Table 4 (see also L242-243): I might have missed it, but have you referenced/justified your CR fitness level classifications (Low, Moderate, High) in the Methods? Please check and add if necessary.

*ANSWER*: We provided the Astorino et al. 2015 reference for the CR classifications in line 226 (line 224 in the revised manuscript).
